# Patients’ and healthcare professionals’ perceived facilitators and barriers for shared decision-making for frail and elderly patients in perioperative care: a scoping review

**DOI:** 10.1186/s12913-023-09120-4

**Published:** 2023-02-24

**Authors:** Amyn Vogel, Camille Guinemer, Daniel Fürstenau

**Affiliations:** 1 School of Business & Economics, Department of Information Systems, Freie Universität Berlin, Berlin, Germany; 2 Charité - Universitätsmedizin Berlin, Corporate Member of Freie Universität Berlin and Humboldt-Universität zu Berlin, Berlin, Germany; 3 Department of Business IT, IT University of Copenhagen, Copenhagen, Denmark

**Keywords:** Shared decision-making, Perioperative care, Barriers and facilitators, Elderly and frail patients, Healthcare innovation implementation

## Abstract

**Background:**

Shared decision-making (SDM) in perioperative care, is an organizational approach to instituting sharing of information and decision-making around surgery. It aims at enabling patient autonomy and patient-centered care. Frail and elderly patients suffering from multiple health conditions and increased surgical vulnerability might particularly benefit from SDM. However, little is known about the facilitators and barriers to implementing SDM in perioperative care for the specific needs of frail and elderly patients.

Our objective is twofold: First, we aim at collecting, analyzing, categorizing, and communicating facilitators and barriers. Second, we aim at collecting and mapping conceptual approaches and methods employed in determining and analyzing these facilitators and barriers.

**Methods:**

The search strategy focused on peer-reviewed studies. We employed a taxonomy which is based on the SPIDER framework and added the items general article information, stakeholder, barriers/facilitators, category, subcategory, and setting/contextual information. This taxonomy is based on preceding reviews. The scoping review is reported under the Preferred Reporting Items for Systematic Reviews and Meta-analyses extension for Scoping Reviews. Based on the databases MEDLINE, Embase, CINAHL, and Web of Science, we screened 984 articles, identified, and reviewed 13 original studies.

**Results:**

Within this review, two primary facilitators concerning patients’ willingness to participate in SDM emerged: Patients want to be informed on their medical condition and procedures. Patients prefer sharing decisions with healthcare professionals, compared to decision-making solely by patients or decision-making solely by healthcare professionals. Communication issues and asymmetric power relationships between patients and clinical healthcare professionals are barriers to SDM. Regarding the methodological approaches, the evaluation of the conceptual approaches demonstrates that the selected articles lack employing a distinct theoretical framework. Second, the selected studies mainly used surveys and interviews, observational studies, like ethnographic or video-based studies are absent.

**Conclusion:**

Diverging findings perceived by patients or clinical healthcare professionals were identified. These imply that SDM research related to elderly and frail patients should become more encompassing by employing research that incorporates theory-based qualitative analysis, and observational studies of SDM consultations for understanding practices by patients and clinical healthcare professionals. Observational studies are particularly relevant as these were not conducted.

**Trial registration:**

https://osf.io/8fjnb/

**Supplementary Information:**

The online version contains supplementary material available at 10.1186/s12913-023-09120-4.

## Contributions to the literature

- This review contributes to the literature on SDM by determining barriers and facilitators specific to frail and elderly patients and perioperative decision-making and by exploring and discussing methodological approaches employed.
- Power and competence asymmetries are at the core of SDM. This is rooted in the belief that only healthcare professionals have the knowledge, expertise and understanding necessary to make decisions.
- The reviewed articles focus on the collection of determinants, enabling or impeding SDM. We suggest a theory-driven analysis, to better understand SDM practices by patients and clinical healthcare professionals.

## Introduction

The age-related demographic change in Western countries and the associated increase in life expectancy result in a steadily growing population of senior citizens. While in 2019 703 million people were aged 65 years or older, this number is projected to increase to 1,5 billion by 2050 [1]. This increase in the number of elderly people who are more susceptible to health problems also poses new challenges for the healthcare system. Thereby, the process of aging is concurrently diverse. This implies significant differences in patient characteristics concerning elderly patients which need to be identified and considered [2].

Specifically, this concerns elderly patients diagnosed with the frailty syndrome within perioperative care. Patients who are affected by frailty are particularly vulnerable. Recent studies indicate that the frailty syndrome, concerning elderly patients, aggravates the health condition and treatment [3, 4]. These patients are subject to multimorbidity, polypharmacy and social isolation, while suffering from symptoms such as cognitive impairment, functional constraints and psychological issues [5–10]. This leads to an increased treatment risk related to postoperative complications [11, 12]. These complications concern increased mortality, prolonged length of stay, decreased quality of life after surgery [13, 14] and postoperative delirium [15], indicating a high level of complexity in surgical procedures for frail patients [16].

Patient autonomy has become a key approach for addressing patient characteristics by empowering patients to communicate on their perceived health condition, needs and requirements [17]. Autonomy refers to the ability of patients to make decisions about their own healthcare and treatment: “Personal autonomy is, at minimum, self-rule that is free from both controlling interference by others and from limitations, such as inadequate understanding, that prevent meaningful choice. The autonomous individual acts freely in accordance with a self-chosen plan [ …]” [18]. In practice, shared decision-making (SDM) represents a pathway to implement patient autonomy, as an alternative organization of decision-making promoting patient participation [19–27]. In this way, SDM empowers patients to exercise their autonomy at a critical moment of care, the decision-making moment, in the sense that personal values and ideas are addressed.

SDM projects are being conducted and regulatory and policy frameworks are being implemented worldwide [28, 29]. At its core, SDM is a decision-making process that embeds collaboration, debate, and responsibility among participating healthcare professionals and patients [21, 22]. SDM is a bridging concept between the information model, which emphasizes patient autonomy, and the paternalistic model, which emphasizes healthcare professionals’ authority [20, 30–32]. There are diverging perspectives for understanding SDM. These perspectives concern the process (how SDM shapes the interactions between patients and healthcare professionals), the objectives (depending on the objectives of the healthcare treatment by the decision-making entities), and the communication (what is being shared and how is it being discussed) [33]. The variations are in part due to the particular settings (i.e., primary care setting or surgical setting) [34], leaving the subject of SDM as conceptually fragmented [33, 35, 36]. The notion of ‘sharing’, by healthcare professionals and patients, is an important factor for these practical variations. Hence, the implementation of SDM ranges from sharing health information, sharing treatment path related information to discussing and sharing the decision-making responsibility [37]. Healthcare practice remains scattered in terms of consistently conceptualizing and implementing SDM in the clinical setting [34–36, 38].

Our understanding of SDM is grounded in Charles et al.’s four pillars [20, 21]. These refer to the (a) participation of at least one patient and one healthcare professional, (b) sharing of all relevant information, (c) willingness to engage in dialogue and compromise, (d) adherence to the treatment decisions taken. Acknowledging patients’ personal needs, conditions, and treatment goals, and the healthcare professionals’ understanding of the patients’ conditions and of potential courses of action are crucial to SDM [39]. Further, SDM requires an element of choice. It therefore concerns elective treatment.

Understanding SDM between patients and healthcare professionals also depends on the setting and the subject of decision. Thereby, perioperative decisions refer to measures and interventions before, during, and after surgery. The subject of decisions involves different issues, treatment pathways, and consequences to consider. Beginning with preoperative decisions, this involves sharing information about the patients’ health condition, considering, for example, pre-existing conditions, regular use of medications, and necessary rehabilitation measures, and how these may affect the surgical procedure. Surgical decisions involve medical necessities, but also patients’ personal preferences, for example, regarding the intervention or anesthesia. Postoperative decisions primarily involve rehabilitative measures in terms of type, location, and anticipated time periods.

### Purpose of this scoping review

While previous reviews [40–43] contribute to capturing and understanding various barriers and facilitators, reviews which are specific to perioperative care and elderly and frail patients are still absent. A search for reviews employing this scope has not yielded any results. The implementation of SDM, into clinical practice, should therefore be studied regarding its facilitators and barriers [23, 28, 44, 45]. The purpose of this research is to review original studies on perceived facilitators and barriers for SDM within perioperative care by elderly and frail patients and clinical healthcare professionals in care of elderly patients.

Although SDM is readily understood as a path to patient autonomy and is associated with positive attributes, the question arises whether this is true for frail and elderly patients and for perioperative care. The implications of perioperative decisions and the complexity of the conditions of elderly and frail patients do not necessarily reflect the conclusions of other reviews. SDM, for elderly and frail patients, demands further research for understanding patient and clinical professionals, who are in care of elderly patients, perceived barriers and facilitators, to improve healthcare treatment within perioperative care.

We conduct a scoping review, to understand the content and the nature of the facilitators and barriers and the underlying methodological approaches. This article pursues the following research questions:

RQ1: What are facilitators and barriers perceived by elderly and frail patients and clinicians for shared decision-making in perioperative care?

RQ2: What are the conceptual approaches and methods used in analyzing facilitators and barriers to the introduction of shared decision-making in perioperative care as perceived by elderly and frail patients and clinicians?

## Methods

The scoping review is reported under the Preferred Reporting Items for Systematic Reviews and Meta-analyses extension for Scoping Reviews (PRISMA-ScR; checklist available in Additional file 1: Appendix 1). We developed a research protocol for this review, which was registered on April 11th, 2022, via the Open Science Framework (https://osf.io/8fjnb/). The methodological framework, procedures, research phases and data charting templates are guided by Arksey and O’Malley [46] and by the Joanna Briggs Institute [47]. Conceptually, the aim of a scoping review is to identify relevant literature on a defined research field. Differentiating features setting scoping reviews apart from systematic reviews concern the research question (broad and specific), study types (all study types and specific study types) and scope of the review (narrow and wide) [46]. We employed the following steps *(i) identifying the research question (ii) identifying relevant studies, (iii) study selection, (iv) charting the data, and (v) collating, summarizing, and reporting the result* [46].

### Identifying the research question

This research phase has been discussed and presented in the introduction of this manuscript.

### Identifying relevant studies

The review was conducted using the databases MEDLINE, Embase, CINAHL, and Web of Science. We did not restrict publication periods. Only English, French, and German language articles were selected, and duplicates were excluded. The databases were searched in February 2022. Following Phelps et al. [48] we first identified three search term subjects, which constitute the basis for the search queries: SDM, the field of SDM application, and patient characteristics. The full search queries are provided in Additional file 2: Appendix 2. Prior to the search, the list of search terms was independently discussed with several specialists in anesthesiology and intensive care medicine, experienced in SDM to avoid crucial omissions.

### Study selection

**Fig. 1:**
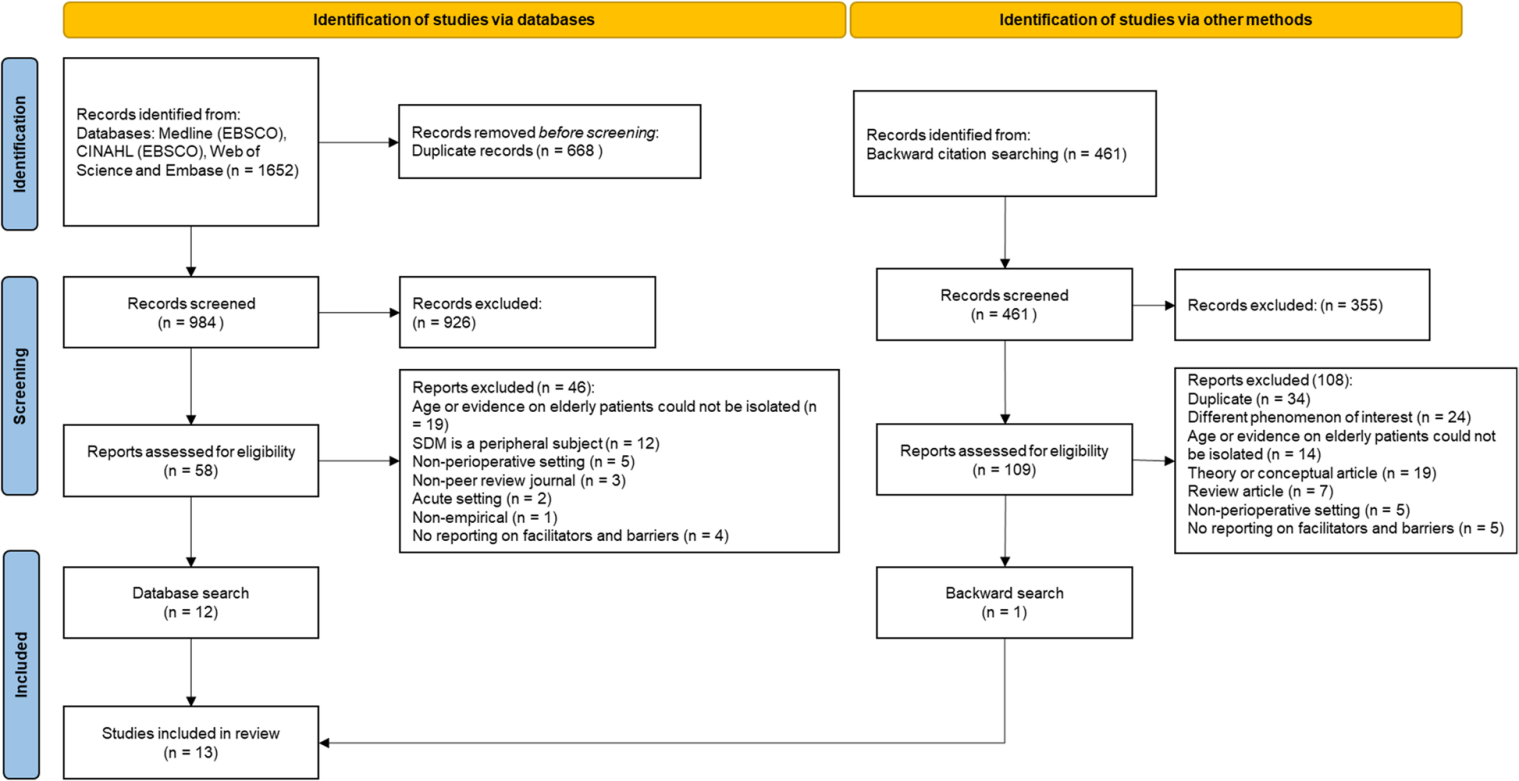
PRISMA: Scoping review process. Based on: Page MJ, McKenzie JE, Bossuyt PM, Boutron I, Hoffmann TC, Mulrow CD, et al. The PRISMA 2020 statement: an updated guideline for reporting systematic reviews. BMJ 2021:n71

The inclusion and exclusion criteria were developed a priori and included in the research protocol. We selected original studies on facilitators and barriers for SDM addressed by elderly and frail patients and clinical healthcare professionals within perioperative care (Fig. 1). We included i) original studies, ii) targeting elderly patients (≥ 65 years), iii) studies being employed in perioperative care, iv) for elective surgery (no acute or emergency setting), v) reporting perceived barriers and/or facilitators to SDM, and vi) studies focusing on either the perspective of patients and/or of clinical healthcare professionals. Clinical healthcare professionals were defined as anesthetists, surgeons and other clinical professions like nurses or physiotherapists related to perioperative care for elderly patients. We also only included peer-reviewed articles. The first selection phase was based on a content analysis of the abstracts and titles of all retrieved articles against the eligibility criteria. Only the articles selected in this phase were chosen for further consideration. In a second selection phase, we screened on full text against the eligibility criteria and retrieved the final selection of articles. We further conducted a backward search, based on the final selection of articles, and screened the title for SDM related content. We employed the respective two selection phases. This study selection has been performed independently by FA (first author) and SA (second author). Subsequently the selection was discussed between FA and SA, until consensus was reached. In cases of persistent discrepancy, respective articles were discussed with LA (last author), until consensus was reached.

### Charting the data and development of data items

In the research protocol we developed a data charting template to organize the data extraction based on the SPIDER framework [49]. This concerns information on the *sample*, *phenomenon of interest*, *design*, *evaluation* and r*esearch type*. We further added the items *article information* (authors, year, country), *stakeholder*, *barriers/facilitators*, *category*, *subcategory*, and *setting/contextual information* as these are relevant for the respective research questions (Additional file 3: Appendix 3).

The item *stakeholder* refers to the concerned stakeholders. Informed by prior reviews [40–43], we identified the following stakeholders: *Patients*, *healthcare personnel*, *decision-making interaction*, and *healthcare system and organization*. *Decision-making interaction* concerns issues affecting the interaction between participants in a SDM conference. *Healthcare system and organization* refers to factors associated with organizational and institutional issues affecting the implementation of SDM. The item *barriers/facilitator* concerns determined factors enabling or impeding SDM. Thereby, we do not conceive of the factors as isolated facilitators and barriers, but rather as a network of factors that are mutually dependent or even mutually exclusive [50]. We believe that this approach enables us to better understand the issues around the implementation of SDM, and the underlying norms, values, and practices of healthcare professionals and patients. The item *subcategory* refers to extracted facilitators and/or barriers. The item *category* refers to thematic clusters of *subcategories*. In a first stage, the selected articles were studied, and relevant sections were extracted. Further, the sections were determined as *facilitators* or *barriers* and *subcategories* were inductively developed. By way of an example: In a text passage we determined the issue of time pressure. This was identified as a barrier and it was documented as a subcategory (‘time pressure’). Following the identification of *subcategories*, we clustered these by subject and inductively developed *categories* (for the respective example: ‘*Treatment Organization and Risk’*). These steps were performed independently by FA and SA and were subsequently discussed between FA and SA. The results and discrepancies were discussed with LA, until consensus was reached. This clustering process is informed by prior reviews [40–43]. The item *setting/contextual information* refers to information on whether SDM consultations took place, and the time of the study in the clinical treatment process (i.e., prior or post-surgical intervention).

### Collating, summarizing, and reporting the result

The extracted information on facilitators and barriers to SDM were organized (charted data for the sources of evidence in Additional file 4: Appendix 4) to provide a comprehensive summary of the results. We further related the *subcategories* to the respective stakeholders in an evidence map (Additional file 5: Appendix 5). FA and SA independently performed the data extraction and charting process and discussed the results with LA, until consensus was reached. FA is a doctoral researcher, working within the field of organization and sociology, conducting research on SDM in perioperative care. SA is a health economics researcher, specialized in healthcare organization. LA is a professor of digitalization and work group leader in healthcare transformation.

## Results

The PRISMA flowchart (Fig. 1) shows the resulting records. A total of 1652 results were retrieved, including 668 duplicates. After screening of titles and abstracts, 58 records were eligible for retrieval and full-text analysis, and 13 were selected for the final analysis. Figure 1 lists the first applying exclusion reason, while several reasons further down the line might apply to an article. Most articles (19) were excluded because either the patient cohort was too young (< 65) or the results for this age cohort could not be separated. Also, SDM is only peripherally mentioned in 12 articles. Other reasons relate to the setting (non-perioperative, five articles), no reporting of facilitators or barriers (four), and no peer-reviewed journals (three). Two articles concern non-elective measures, in emergency settings, and one article is a theoretical discussion, without an empirical study. Building upon the 13 selected articles, we performed a backward search for references [51, 52] to reduce database or search term related omissions. We thereby screened the titles of 461 articles for SDM related content. We further selected 109 articles for full text analysis and after applying the exclusion criteria we selected one further article for the final analysis.

### Characteristics of studies included in the scoping review and methodological considerations

The 13 studies included in this review were published between 2006 and 2021 and eight were conducted in the United States. The remaining six studies have been conducted in Canada, Netherland (2), Norway, Sweden, and Switzerland.

On the cohort characteristics: Nine articles exclusively address patients, and three explore patients, surgeons, and other clinical employees, and one concerns surgeons (Table 1). The cohort size ranges from 11 participants to 718. Overall, the studies concern varying diagnoses and surgical treatments, albeit three articles exclusively concern female patients, addressing breast cancer. Hereby, elective surgery constitutes the common denominator. Further, four articles explicitly concern frail patients.

**Table 1:** Characteristics of selected articles

First author, year of publication, country of origin	Cohort size	Frailty	Age and condition	Study setting: Did the study take place prior to or after a surgical intervention?	Study setting group
Aasen et al., 2012, Norway [65]	11 patients	Y	Age:72–90 Condition: End-stage renal disease	Post surgery	HYP
Barrett et al., 2021, US [53]	447 patients	N	Age: Median 72 (64–80) Condition: Chronic kidney disease	Prior to surgery	PRE
Bleicher et al., 2008, US [54]	1259 (571) patients	N	Age: 61–70; > 70 Condition: ductal carcinoma in situ (DCIS) invasive breast cancer, comorbidities	Post-surgery (1–14 months)	HYP
Dardas et al., 2016, US [55]	99 patients	N	Age: ≥ 65 Condition: Joint-arthritis	Prior to surgery	PRE
Deme et al., 2021, US [56]	6 patients 5 spine surgeons	Y	Age: ≥ 65 Condition: Adult spinal deformity	Post surgery	HYP
De Roo et al., 2021, US [57]	46 surgeons	N	Concerning age: ≥ 65,	No surgical setting	HYP
Ekdahl et al., 2010, Sweden [58]	15 patients	Y	Age: ≥75 Condition: Various diagnoses (i.e. COPD, hypertonia, malignant melanoma, aortic insufficiency), frail	Prior to surgery	HYP
Gainer et al., 2017, Canada [59]	15 patients 13 nurses, physiotherapists and occupational therapists 5 intensive care nurses 12 surgeons, anesthetists and cardiac intensivists	Y	Age: ≥65, mean: 74,9 Condition: Patients referred to cardiac surgery	Post surgery (within eight weeks, eight weeks and after two years) complicated post-op course	HYP
Hamelinck et al., 2018, Netherlands [60]	74 patients (34% ≥65 years)	N	Age: ≥ 65 Condition: DCIS; invasive tumor	Prior to and post surgery	PRE/POST
Huetteman et al., 2018, US [61]	30 patients	N	Age: ≥62 Condition: Distal radius fracture	Post surgery	POST
Mandelblatt et al., 2006, US [62]	718 patients 38 related surgeons	N	Age: ≥67 Condition: invasive breast cancer	Post surgery	POST
Uldry et al., 2013, Switzerland [63]	253 patients (^a^)	N	Age: median 58,3 (“old” patients ≥65) Condition: N/A	Prior to surgery	HYP
Verberne et al. 2019, Netherlands [64]	99 patients	N	Age: ≥70 Condition: Stage 4/5 chronic kidney disease	Post-surgery	POST

^a^The exact number of patients aged 65 or older could not be determined

All articles reported on facilitators and barriers. However, the studies reported predominantly on barriers (37) compared to facilitators (23).

For considerations on the setting of the studies, we considered two aspects: (A) Did the study implement SDM consultations? And (B) Did the study take place before and/or after surgical treatment? For (A) we considered whether the studies implemented SDM consultations and whether these studies have been conducted before and/or after the consultation (Table 1). Based on these considerations we created four types of categories: Studies without SDM consultations (HYP; hypothetical), studies with SDM consultations prior to (PRE), after (POST) or prior and after the study (PRE/POST).

Six studies were concerned with SDM consultations as their object of study [53, 55, 60–62, 64]. Two of these conducted the study before the consultation, three studies were conducted after the consultation and one article refers to conducting the study before and after the SDM consultation. The remaining studies (7) did not implement SDM consultations. The four studies considering frail patients did not implement SDM consultations.

For (B), four studies assessed patients preferences for involvement prior to a surgery [53, 55, 58, 63]. Most studies (7) were conducted as a follow-up with patients after an intervention. One article conducted the study before and after the surgery [60], and one study did not conduct the study around surgery [57].

Regarding the methodological approach, we considered information on the *design*, *evaluation*, and *research type* (Table 2). Quantitative and qualitative approaches were found (seven and five). The quantitative approaches either used established questionnaires and scales (three articles) (i.e., SDM-Q-10, SDM-Q-Doc, or Control Preference Scale) or generated their own items for the study (four articles). The qualitative approaches mainly used semi-structured interviews for gathering data, but employed different analytical approaches: Discourse analysis [65], content analysis based on Graneheim and Lundman [58], thematic analysis [56, 57, 59], and grounded theory [61].

**Table 2:** Methodological approaches

Author	Design		Evaluation	Research type		
	Intervention	Control group	Evaluation	Data collection	Measurement point	Analysis method
Aasen, 2012 [65]	No	N/A	Patients’ perception of participation and SDM	Interview (open-ended)	Cross-sectional	Qualitative analysis (inductive, critical discourse analysis)
Barrett, 2021 [53]	Yes	No	Patient preferences and experiences with SDM	Survey (control preference scale)	Cross-sectional	Statistical analysis (logistic regression)
Bleicher, 2008 [54]	No	N/A	Age-related preferences on SDM	Survey (online and telephone, 143 questions)	Cross-sectional	Statistical analysis (logistic regression)
Dardas, 2016 [55]	Yes	No	Patient preferences on SDM	Survey (control preference scale)	Cross-sectional	Statistical analysis (descriptive statistics)
Deme, 2021 [56]	No	N/A	Patients and spinal surgeons perception on SDM	Interview (semi-structured)	Cross-sectional	Qualitative analysis (inductive and deductive content analysis)
De Roo, 2021 [57]	No	N/A	Surgeons’ perception on participation and SDM;	Interview (semi-structured)	Cross-sectional	Qualitative analysis (inductive content analysis)
Ekdahl, 2010 [58]	No	N/A	Patients’ preferences on participation and SDM	Interview (semi-structured)	Cross-sectional	Qualitative analysis (inductive content analysis based on Graneheim and Lundman)
Gainer, 2017 [59]	No	N/A	Patients and healthcare personnel perception on decision-making and SDM	Interview (semi-structured, focus group)	Longitudinal	Qualitative analysis (inductive content analysis)
Hamelinck, 2018 [60]	Yes	No	Age-related patient preferences and experiences on SDM	Survey (modified control preference scale)	Cross-sectional	Statistical analysis (descriptive statistics)
Huetteman, 2018 [61]	Yes	Yes	Patients’ perceived and desired role of healthcare providers in decision-making	Interview (semi-structured)	Cross-sectional	Qualitative analysis (inductive content analysis, grounded theory based)
Mandelblatt, 2006 [62]	Yes	No	Treatment related outcomes of SDM	Interview (semi-structured)	Cross-sectional	Statistical analysis (logistic regression and random effects panel regression)
Uldry, 2013 [63]	No	N/A	Patient preferences for decision-making	Survey (binary questions, 13 questions)	Cross-sectional	Statistical analysis (logistic regression and linear regression)
Verberne et al. 2019 [64]	Yes	No	Patient preferences and experiences with SDM	Survey (binary questions11-point Likert scale and open-ended questions)	Cross-sectional	Statistical analysis (descriptive statistics)

### Perceived facilitators and barriers by patients and clinical healthcare professionals

. We identified 37 barriers and 23 facilitators, which are clustered in five *categories: Attitude and behavior*, *trust and power*, *knowledge and communication*, *treatment organization and risk*, and *health and age*. Table 3 provides an overall picture of the identified *categories*. It also provides an overview of the identified facilitators and barriers and the associated number of articles in which these were discussed. A table with definitions of each facilitator and barrier is attached in Additional file 6: Appendix 6.

**Table 3:** Empirical results on barriers and facilitators

Category	Stakeholder	Facilitators (Nr. of articles)	Barriers (Nr. of articles)
**Attitude and Behavior**	Patient factors	Active behavior (9)	Depending on family or healthcare personnel (6)
		Confidence in participating in decisional involvement (3)	Not having a choice (4)
		Wanting to be informed or demanding more information (7)	Not wanting to participate in decision-making (4)
		Wanting to be involved through decisional participation (9) Being enabled to ask questions and make decisions (2)	Passive behavior (6)
			Submissive behavior (3) Being overstrained (2) Discomfort due to too much involvement (1)
	Healthcare personnel factors		No decisional involvement of patients (6)
			No treatment or information involvement of patients (5)
**Trust and Power**	Patient factors	Exercising power and dominance (6)	Asymmetric power relationship (4)
		Unknown healthcare provider (3)	Fear of incompliance (1)
			Feeling controlled (1)
			Feeling incapacitated (2)
			Feeling powerless / Having no control (3)
			Institution of power and/or trust (1)
			Passive behavior (6)
			Submission towards healthcare personnel (3)
			Submissive communication semantics (2)
			Submissive behavior (3)
			Trust towards healthcare personnel (4)
			Unknown healthcare provider (4)
	Healthcare personnel factors		Asymmetric power relationship (4)
			Exercising power and dominance (5)
			No treatment or information involvement of patients (5)
	Decision-making interaction factors		Asymmetric power relationship (4)
			Trust towards healthcare personnel (4)
			Unknown healthcare provider (4)
**Knowledge and Communication**	Patient factors	Adequate medical knowledge (1)	Depending on family or healthcare personnel (6)
		Being offered a choice (1)	Ease of non-involvement (1)
		Internet as source for medical information (2)	Knowledge/Competence asymmetry (5)
		Medical knowledge is not required (2)	Lack of medical or treatment related knowledge (5)
		Supporting family involvement (1)	Prior misinformation through family, friends, internet or other sources (1)
		Wanting to be informed or demanding more information (7)	Submissive communication semantics (2)
		Wanting to be involved through decisional participation (9)	
		Wanting to express themselves, issue opinions and preferences and to be heard (6) Satisfying information sharing (3)	
	Healthcare personnel factors	Comprehensive communication (1)	Dominant communication semantics (2) Linguistic issues (3) No treatment or information involvement of patients (5)
	Decision-making interaction factors		Diverging perceptions of health condition, treatment or surgical outcome (1)
			Knowledge/Competence asymmetry (5)
	Healthcare system /organizational factors		No treatment or information involvement of patients (5)
**Treatment Organization and Risk**	Patient factors	Alternative choices / Ambiguity (2) Satisfying information sharing (3) Treatment satisfaction (3) Satisfying involvement (2) Diminution of decisional conflict (1)	Facing different treatment strategies (1)
			Timely treatment necessity (2)
			Treatment related dismissal of decisional involvement (5)
	Healthcare personnel factors		Facing different treatment strategies (1)
			Time pressure (5)
	Decision-making interaction factors	SDM mediator (1)	Treatment related dismissal of decisional involvement (5)
			High workload (3)
	Healthcare system /organizational factors	Adequate workload (1) Formal SDM approach (1)	Acute setting (1)
			Healthcare staff rotation (2)
			High Workload (3)
			Lack of integration in social practices (3)
			Patient turnover (1)
			Scheduling issue (1)
**Health and Age**	Patient factors		Being ill (2)
			Being in pain (2)
			Being old (4)
			Being overstrained (3)
			Being tired (1)
			Forgetting discussions or given information (2)
			Timely treatment necessity (2)
			Discomfort due to too much involvement (1)
	Healthcare system /organizational factors	Need for individualized care - Treatment complexity and multimorbid patients (3)	

It should also be noted that this category system aims to cluster the identified factors as accurately as possible. However, we are also aware that there are some issues related to implementing a category system. This applies above all to the categories “knowledge and communication” and “trust and power”. This refers to, for example, the implication that knowledge asymmetries have an impact on power asymmetries and that communication has an influence on trust issues. In this sense, the categories are thus not to be understood as isolated, but as being in relation to other categories and their underlying factors.

Lastly, we mapped the concerned stakeholders to the assessed facilitators and barriers. A short version of these results is also included in Table 3, the full version is attached in Additional file 5: Appendix 5.

**Fig. 2:**
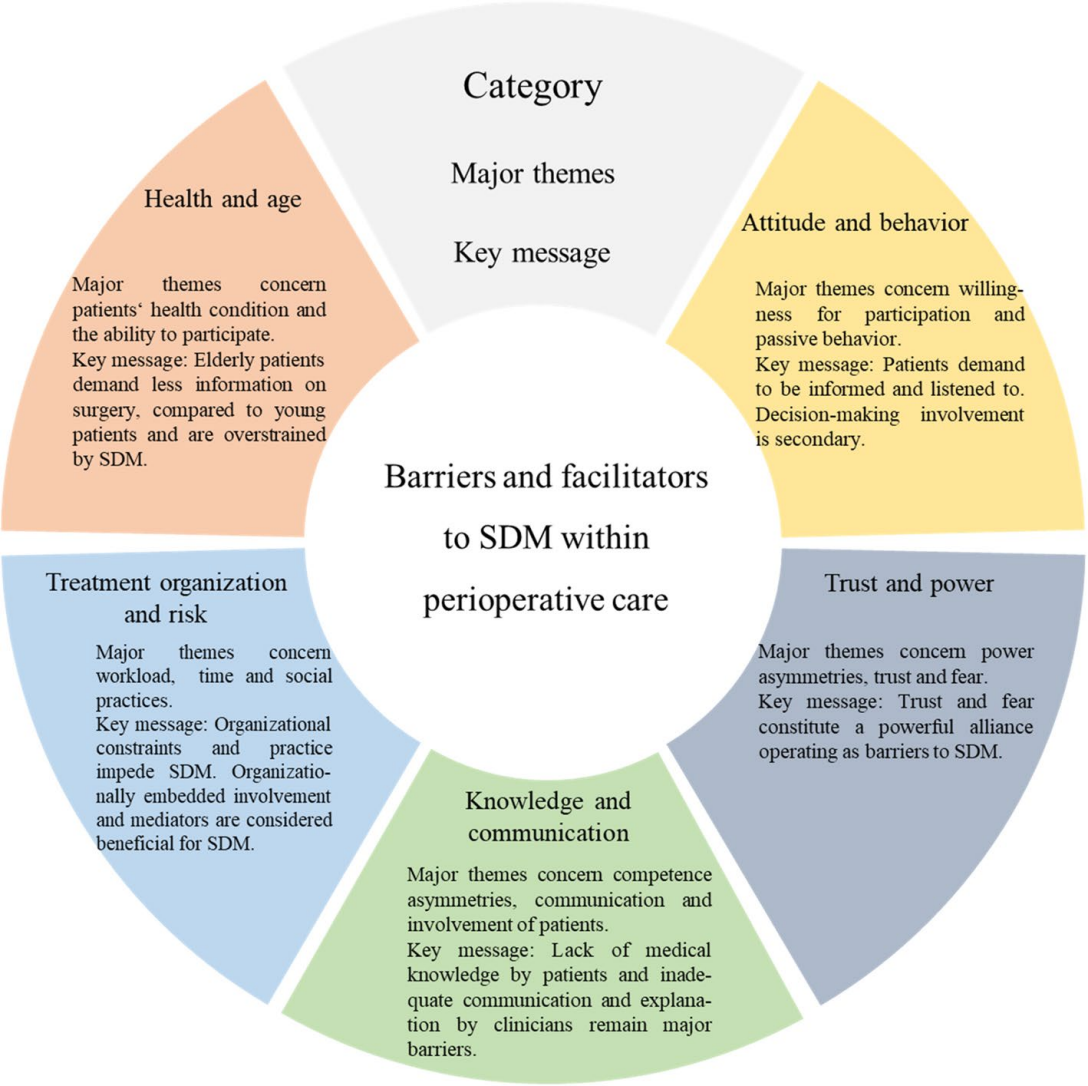
Categories and primary subjects

Figure 2 summarizes the main insights from the analysis on facilitators and barriers. It offers an overview of the applied *categories*, main subjects, and the key message.

### Attitude and behavior

This *category* highlights the contrast between the will and desire and the rejection of participation by patients and implies further factors related to patients’ and healthcare professionals’ attitudes and behavior.

Major barriers in this *category* concern the lack of involvement of patients by healthcare professionals [56–59, 65], lack of confidence in participating [46–48] and the sense of not having a choice [56, 59, 61, 64]. Issues related to passive and submissive behavior by patients are further barriers [36, 42–45, 47].

As facilitators for SDM, the will to be informed and also to participate in decisions is mentioned in most of the articles [54–56, 58–61, 63, 65]. These studies demonstrate the importance for patients to be treated with respect and to be listened to. Likewise, being informed and receiving explanations is considered essential by patients [59, 63].

#### Trust and power

This *category* mainly concerns perceived power asymmetries and related trust issues.

Asymmetric power relationships were referred to as barriers to SDM [58, 59, 65]. The dominance of healthcare professionals and power asymmetries are a particularly hindering factor, as patients perceive the hospital as an institution of power, not only in terms of its authority and hierarchy, but also regarding healthcare professionals to whom competence and responsibility for patient care are attributed [58]. Patients not only deny themselves any competence vis-à-vis clinical decision-making, and attribute full competence to healthcare professionals, but also fear that the success of the therapy might be compromised, if they did not comply with the treatment proposed by healthcare professionals [65]. This would even lead to patients agreeing to treatments, although believing that this might interfere with their specific requirements and quality of life [65]. In one study, patients even preferred a “computer pick an option for them at random” [61] over their own participation in decision-making.

Anxiety and the feeling of being controlled are further significant barriers. Patients believe that they have to adapt, eliminate doubts and follow the professional opinion of healthcare professionals in order to receive good medical treatment [58, 59, 65].

The extent of familiarity with the attending healthcare professionals also affects preferences for participation: For patients with multiple visits, who know the concerned healthcare professionals, patients tend to prefer SDM, while for unfamiliar healthcare professionals, patients tend to prefer a leading role [53, 55].

Exercising personal autonomy is in turn conceived as an essential facilitator for SDM by patients [47].

### Knowledge and communication

This *category* centers on the asymmetry of knowledge and competence between healthcare professionals and patients and related communicational issues [58, 59, 61, 65].

The main barrier concerns the attribution of competence and knowledge required for decisions on the treatment to healthcare professionals, while patients are considered to lack these competences [58, 59, 65]. Thereby, medical competence becomes the sole significant attribute, without considering personal needs, requirements and preferences of patients. This is in line with a study that examined factors influencing surgical decisions for high-risk patients from surgeons’ perspective. The study shows that surgical experience is given considerable weight compared to patient opinions, requirements and preferences, which remain underrepresented [57]. Inadequate communication on health conditions and procedures and non-explanation of post-operative outcomes accentuate competence asymmetries as barriers to patient participation for decision-making and therapy adherence [56, 59]. Mutual willingness to engage in dialogue and clear communication on health conditions and treatment procedures are considered to be facilitators [40–43, 48]. However, in an age-comparative study, the imperative of the relationship between competence and decision-making authority was decoupled: Perceived health competence did not imply a different approach towards decisional participation [54, 55]. Although elderly women report lower health competence than younger women, this has no relevant influence on the will to participate [54].

### Treatment organization and risk

This *category* centers on organizational factors.

Predominantly positive aspects were associated with SDM. This refers to patient involvement and explanation of treatment procedures [54, 55, 58, 62, 63, 65].

Barriers relate foremost to the workload and time pressure to which healthcare professionals are subject [55, 57–59, 65]. Further barriers concern aspects such as heterogeneity of involved healthcare professionals [58], staff rotation, diverging treatment strategies [58, 59] and lack of integration of SDM in clinical practices [53, 57, 65].

The surgical setting represents a barrier for conceiving the possibility of alternative decision-making options: Patients believed that surgery was the only viable option and that this decision must be taken urgently, thus leading to the perception of time constraints [56, 64].

In turn, organizationally embedded SDM consultations are considered to be SDM facilitating [47]. The implementation of a mediator, someone who does not take any decisions and only serves the purpose of building a communicative bridge between the participants, is perceived as beneficial for the successful organization of SDM [47].

### Health and age

This category mainly concerns health related issues and predominantly concerns studies referring to frail patients.

Major barriers relate to age, health status, and patients being overwhelmed [57–59, 61, 62, 64, 65]. In particular, age-related studies were performed: The primary subject was to conduct age-related analysis on preferences and perceived participation perioperative decision-making. Overall, no relevant age-related differences concerning the preferences towards SDM were found [54, 60]. It was also found that patients prefer an active role (sole decision or shared) [60]. A difference regarding decisional participation was identified concerning specific interventions [60].

In an age-comparative study, it was also shown that elderly patients demand less information than younger patients and thus rely more on surgical opinion. Also, elderly patients are more likely to hand over the responsibility of decision-making to healthcare professionals than younger patients [63]. Further, it was found that patients who were more involved than they preferred had a higher risk of doubting the intervention and being overstrained [54]. Still, if desired by patients, SDM could lead to an increased sense of control and short-term satisfaction [62].

## Discussion

Through this scoping review we reviewed original studies on facilitators and barriers for SDM within perioperative care perceived by elderly and frail patients and clinical healthcare professionals in care of elderly patients. We further reviewed the employed approaches and methods.

### Facilitators and barriers

Considering the attitude towards and willingness for SDM by patients, we determined a set of diverging findings: While some studies suggest that elderly patients are eager to participate, in other studies patients even prefer ‘decision-making’ by a computer, over their own. This review determines trust and fear as constitutive factors for patients preferring the opinion of healthcare professionals and a paternalistic approach, leading to the expectation that healthcare professionals should at least initiate and lead the discussions or make recommendations. Trust and fear therefore constitute a powerful alliance impeding patient participation. Although this has also been discussed in previous reviews [40, 42], these factors hold a more prominent role in this review. Particularly for patients, the unfamiliar role as a patient, language barriers, and vulnerability prior to surgery contribute to these patients’ hopes for redemption and accentuate the alliance of trust and fear.

This review finds power and knowledge asymmetries at the core of participation. These issues are rooted in the belief that only healthcare professionals have the knowledge, expertise and understanding of the patients’ condition necessary to make decisions. The responsibility and decision-making authority are attributed to them. Although this has also been discussed in previous reviews [40–42], this review offers insights to issues substantiating these asymmetries, particularly concerning elderly patients. This is related to traditional role assumptions and the understanding of surgical interventions as exclusively medical issues, without considering personal needs and preferences. This perception and the associated understanding of roles shape the relationship between patients and healthcare professionals. Changing these attitudes is a crucial aspect to embrace patient involvement in decision-making. In particular, if we consider some of the complex issues, like perceived power and competence asymmetries, which have come to light in this review, it is necessary to explore the rationales and relations between facilitators and barriers. What are the core believes underpinning those facilitators and barriers? And what do they tell us about the role of patients, the role of healthcare professionals, and the ingredients of their relation? At this point, further studies are needed to better understand the landscape of SDM within a perioperative care for frail and elderly patients.

Regarding frail patients, there are no substantial differences across the studies. The only *category* which primarily concerns studies involving frail patients is *health and age*. The sense of being overwhelmed, tired, or confused mainly concerns frail patients. Since this review revealed that only four studies concern frail patients, more studies are required to substantiate or extend these results. Further, the studies on frail patients did not conduct formally embedded SDM consultations. We therefore argue for the need to conduct more studies explicitly concerning frail patients embedded in SDM consultations.

### Methodological considerations

The analysis of the methodological approaches demonstrates an equal distribution of quantitative and qualitative approaches. It should be emphasized that the qualitative approaches exhibit a methodological heterogeneity. While these cannot be easily compared (in contrast to the use of the established quantitative questionnaires and surveys), these articles offer more in-depth analysis (i.e., asymmetric power relationship and dominance were solely explored by these articles). Further, while in the reviewed studies diverging approaches were employed, observational studies (i.e., non-participatory observations or videographic studies of SDM consultations) [66] of SDM consultations did not take place. Thus, we only know how SDM is *theoretically* dealt with, but not how it is *practically* addressed in a SDM consultation by its’ participants. The actions, conversations, and proceedings of SDM consultations remain omitted. Given diverging approaches in practice [33–38], we consider observational studies of SDM consultations as significant.

### Reflection and outlook

Our analysis on the employed approaches and methods suggests that SDM research concerning elderly and frail patients should become more encompassing by employing further qualitative studies, and observational studies of the SDM consultations. The evaluation of the conceptual approaches demonstrates that the selected articles are primarily concerned with the collection of determinants that enable or impede the implementation of SDM, without employing a distinct theoretical framework. Thus, the articles deprive themselves of the possibility for a deeper, theory-driven analysis of the determinants, on their scope, discussion of causes, inherent relationships, and implications. We believe this to be important, since SDM is scattered in its theoretical embedding and practical approaches [33–38], leading to diverging emphasis and understanding of its’ implementation and relevant issues. By way of example, the issue of asymmetric power relation between patients and healthcare professionals is not a factual entity, determined and deemed relevant by any study. Rather, its’ observable elements are contingent to interpretation, and therefore shaped by the ones perceiving its’ elements. A theory-based approach might pave the road to a distinct, coherent, and profound analysis and interpretation of the issue – here, Aasen et al. [65], Ekdahl et al. [58] and Gainer et al. [59] already provide valuable insight for a deeper analysis of this very subject. Based on Bourdieus’ oeuvre on power and language, Nimmon and Stenfors-Hayes [67] offer an exemplary contribution for a theory-based analysis on power in the patient-physician relationship.

In light of this, we suggest theory-driven, qualitative studies that examine facilitators and barriers of clinical healthcare professionals and patients, in combination with an observational study of SDM consultations. The study of practice is in turn indispensable to enable a thorough understanding of the phenomenon [66, 68, 69].

### Limitations

The reviewed studies exhibit varying degrees of information on methods employed and setting of the study, resulting in limitations on drawing generalizable conclusions. Only four studies addressed frailty. Thus, the results refer mainly to elderly patients. However, the absence of studies on frailty justifies the need to conduct further empirical studies explicitly focused on frailty. Initially, we included the frailty syndrome as a prerequisite, next to elderly patients, in the selection process. A preliminary test of the inclusion and exclusion criteria, described in the protocol, indicated a lack of studies on frailty. We therefore decided not to implement the frailty syndrome as a prerequisite, but to screen for it in selected studies. The qualitative analysis of the articles implies subjective activities, shaped by the background of the researchers. We have tried to minimize this limitation by providing an accountable taxonomy, conducting separated analysis before discussing and employing methodological transparency.

## Conclusion

This scoping review aims at providing a comprehensive overview of original studies on perceived facilitators and barriers for SDM within perioperative care by elderly and frail patients and clinical healthcare professionals, in care of elderly patients. Following the Preferred Reporting Items for Systematic Reviews and Meta-analyses extension for Scoping Reviews process we selected 13 articles for a qualitative analysis.

The results to date imply a plethora of diverging findings, facilitators and barriers perceived by patients or clinical healthcare professionals for SDM in perioperative care for elderly and frail patients. While some studies clearly demonstrate that patients want to actively participate in decision-making processes, regardless of their perceived health competence, other studies suggest that decision authority and competence asymmetries are crucial barriers. These relate to the critical alliance of patients’ fear towards decision-making and trust in healthcare professionals, constituting a preference for a paternalistic approach. Underlying asymmetrical competence and power relationships require further exploration. Healthcare professionals tend to be receptive to SDM, whereas patients’ lack of health competence and structural aspects (time and organization) remain barriers to them. At this point, further studies are needed to better understand the landscape of SDM in a perioperative care for frail and elderly patients.

The examined articles concern primarily the collection of determinants that enable or impede the implementation of SDM. We suggest a theory-driven analysis of the determinants. We further recommend conducting observational studies of actual SDM consultations, to better understand SDM practices by patients and clinical healthcare professionals. As the selected studies only used surveys and interviews, the actual process of patient involvement in decision-making remains omitted.

## Supplementary Information

**Additional file 1: Appendix 1.** Preferred Reporting Items for Systematic reviews and Meta-Analyses extension for Scoping Reviews (PRISMA-ScR) Checklist.

**Additional file 2: Appendix 2.** Full Search Query.

**Additional file 3: Appendix 3.** Data extraction template.

**Additional file 4: Appendix 4.** Data Charting.

**Additional file 5: Appendix 5.** Collated Categories, Subcategories and Stakeholders.

**Additional file 6: Appendix 6.** Taxonomy of barriers and facilitators.

## Data Availability

The datasets supporting the conclusions of this article are included within the article and its additional files.
