# Supplementary material for: Patients’ and healthcare professionals’ perceived facilitators and barriers for shared decision-making for frail and elderly patients in perioperative care: a scoping review
Source: BMC Health Serv Res. 2023 Feb 24;23:197. doi: 10.1186/s12913-023-09120-4 (PMC9960423; doi:10.1186/s12913-023-09120-4)
Supplement: Supplementary file 2 — Additional file 2: Appendix 2. Full Search Query. [file 12913_2023_9120_MOESM2_ESM.docx]

**Appendix 2: Full Search Query**

| **Search Engine and Date** | **Query Text** |
| --- | --- |
| EBSCO Host - MEDLINE and CINAHL  24.02.2022  Applied to: Titles and abstracts  Limits:  only English, French and German language articles | TOPIC:  (  (shared decision making OR  shared decision-making OR  sdm)  AND  (perioperative OR  peri-operative OR  pre-operative OR  preoperative OR  surgical OR  surgery OR  clinic)  AND  (frailty OR  frail OR  elderly OR  OR elder  OR aged  OR older)  ) |
| WebofScience  24.02.2022  Applied to: Titles and abstracts  Limits:  only English, French and German language articles | (  (shared decision making OR  shared decision-making OR  sdm)  AND  (perioperative OR  peri-operative OR  pre-operative OR  preoperative OR  surgical OR  surgery OR  clinic)  AND  (frailty OR  frail OR  elderly OR  OR elder  OR aged  OR older)  ) |
| Embase  24.02.2022  Applied to: Titles and abstracts  Limits:  only English, French and German language articles | (  (shared decision making OR  shared decision-making OR  sdm)  AND  (perioperative OR  peri-operative OR  pre-operative OR  preoperative OR  surgical OR  surgery OR  clinic)  AND  (frailty OR  frail OR  elderly OR  OR elder  OR aged  OR older)  ) |
