# Supplementary material for: Patients’ and healthcare professionals’ perceived facilitators and barriers for shared decision-making for frail and elderly patients in perioperative care: a scoping review
Source: BMC Health Serv Res. 2023 Feb 24;23:197. doi: 10.1186/s12913-023-09120-4 (PMC9960423; doi:10.1186/s12913-023-09120-4)
Supplement: Supplementary file 3 — Additional file 3: Appendix 3. Data extraction template. [file 12913_2023_9120_MOESM3_ESM.docx]

| **Appendix 3: Data extraction template** | |
| --- | --- |
| Article information | General information (authors, year of publication, and country) |
| Sample | Number patients and characteristics |
| Phenomenon of interest | Objective of the selected study |
| Design | Methodological design and study procedures |
| Evaluation | Reporting items |
| Research Type | Employed data collection, measurements and analysis approaches |
| Setting and contextual information | Information on the setting of the selected study. A) Whether the study implemented SDM consultations, if the study has been conducted before or after the SDM consultation, and if the study has been conducted before or after perioperative measures |
| Stakeholder | Determined stakeholders: Patients, healthcare personnel, decision-making interaction, and healthcare system and organization |
| Subcategory | Extracted issue (i.e., time pressure and workload), inductively developed, informed by prior reviews [46,48,49,53] |
| Category | Clustered subcategories (i.e., healthcare treatment and organization), inductively developed, informed by prior reviews [46,48,49,53] |
| Barrier/Facilitator | Indication if subcategory is determined as barriers or facilitators to SDM |
