# Supplementary material for: Patients’ and healthcare professionals’ perceived facilitators and barriers for shared decision-making for frail and elderly patients in perioperative care: a scoping review
Source: BMC Health Serv Res. 2023 Feb 24;23:197. doi: 10.1186/s12913-023-09120-4 (PMC9960423; doi:10.1186/s12913-023-09120-4)
Supplement: Supplementary file 4 — Additional file 4: Appendix 4. Data Charting. [file 12913_2023_9120_MOESM4_ESM.docx]

**Appendix 4: Data Charting**

| Author | Stakeholder | Barrier/Facilitator | Category | Subcategory | Quote |
| --- | --- | --- | --- | --- | --- |
| Aasen et al. | Decision-making interaction factors | Barrier | Trust and Power | Asymmetric power relationship | The first and dominant discourse was called the health-care team’s power and dominance. Both environmental conditions and the team’s practice exercised power and control over the patients. The patients trusted the health-care team, but some felt powerless and were afraid of what might happen if they refused to follow the instructions. The health-care team owned the knowledge and decided what the patients needed to know |
| Aasen et al. | Healthcare personell factors | Barrier | Trust and Power | Exercising power and dominance | The first and dominant discourse was called the health-care team’s power and dominance. Both environmental conditions and the team’s practice exercised power and control over the patients. The patients trusted the health-care team, but some felt powerless and were afraid of what might happen if they refused to follow the instructions. The health-care team owned the knowledge and decided what the patients needed to know |
| Aasen et al. | Patient factors | Barrier | Trust and Power | Trust towards healthcare personnel | The patients trusted the health-care team, but some felt powerless and were afraid of what might happen if they refused to follow the instructions. |
| Aasen et al. | Patient factors | Barrier | Trust and Power | Fear of incompliance | The patients trusted the health-care team, but some felt powerless and were afraid of what might happen if they refused to follow the instructions. |
| Aasen et al. | Patient factors | Barrier | Knowledge and Communication | Knowledge/Competence asymmetry | The health-care team owned the knowledge and decided what the patients needed to know |
| Aasen et al. | Healthcare system /organisatonal factors | Barrier | Treatment Organization and Risk | Lack of integration in social practices | The elderly patients’ right to participate in their haemodialysis treatment did not seem to be well incorporated into the social practices of haemodialysis units. |
| Aasen et al. | Healthcare personell factors | Barrier | Trust and Power | Exercising power and dominance | The health-care team’s power in the dialysis units was expressed in the patients’ stories. This dominance – the power in the interaction between the health-care team and the patients – was apparent not only in the modality but also in the use of appraisal words and metaphors. |
| Aasen et al. | Decision-making interaction factors | Barrier | Knowledge and Communication | Dominant communication semantics | This dominance – the power in the interaction between the health-care team and the patients – was apparent not only in the modality but also in the use of appraisal words and metaphors. The patients often used words like ‘professionals decide’ and ‘always’ when talking about the health-care team. |
| Aasen et al. | Healthcare personell factors | Barrier | Knowledge and Communication | Dominant communication semantics | This dominance – the power in the interaction between the health-care team and the patients – was apparent not only in the modality but also in the use of appraisal words and metaphors. The patients often used words like ‘professionals decide’ and ‘always’ when talking about the health-care team. |
| Aasen et al. | Decision-making interaction factors | Barrier | Knowledge and Communication | Submissive communication semantics | When the patients talked about themselves, they used words like ‘must’, ‘should’, ‘accept’ and ‘trust’, which might say something about the patients’ sense of powerlessness. |
| Aasen et al. | Patient factors | Barrier | Knowledge and Communication | Submissive communication semantics | When the patients talked about themselves, they used words like ‘must’, ‘should’, ‘accept’ and ‘trust’, which might say something about the patients’ sense of powerlessness. |
| Aasen et al. | Healthcare personell factors | Barrier | Trust and Power | Asymmetric power relationship | The first and dominant discourse was called the health-care team’s power and dominance. Both environmental conditions and the team’s practice exercised power and control over the patients. The patients trusted the health-care team, but some felt powerless and were afraid of what might happen if they refused to follow the instructions. The health-care team owned the knowledge and decided what the patients needed to know |
| Aasen et al. | Patient factors | Barrier | Trust and Power | Asymmetric power relationship | When the patients talked about themselves, they used words like ‘must’, ‘should’, ‘accept’ and ‘trust’, which might say something about the patients’ sense of powerlessness. |
| Aasen et al. | Patient factors | Barrier | Trust and Power | Asymmetric power relationship | Several patients used metaphors to describe their feelings. They referred to the dialysis unit as a ‘jail’, the contact nurse as ‘the guardian’, the machine as ‘the animal’ and themselves as ‘furniture’ or ‘a package’. All these metaphors describe a context in which the health-care team has power and control. |
| Aasen et al. | Patient factors | Barrier | Knowledge and Communication | Submissive communication semantics | Several patients used metaphors to describe their feelings. They referred to the dialysis unit as a ‘jail’, the contact nurse as ‘the guardian’, the machine as ‘the animal’ and themselves as ‘furniture’ or ‘a package’. All these metaphors describe a context in which the health-care team has power and control. |
| Aasen et al. | Patient factors | Barrier | Knowledge and Communication | Submissive communication semantics | The use of ‘one’ instead of ‘I’ might show that the healthcare team’s dominance was difficult to talk about and that the patient wanted to distance herself from this theme. |
| Aasen et al. | Patient factors | Barrier | Attitude and Behaviour | Passive behaviour | She missed engaging in dialogue and shared knowledge with the health-care team. As a consequence, she will probably be passive and feel resigned. |
| Aasen et al. | Healthcare personell factors | Barrier | Knowledge and Communication | Dominant communication semantics | She missed engaging in dialogue and shared knowledge with the health-care team. As a consequence, she will probably be passive and feel resigned. |
| Aasen et al. | Healthcare personell factors | Barrier | Attitude and Behaviour | No decisional involvment of patients | She missed engaging in dialogue and shared knowledge with the health-care team. As a consequence, she will probably be passive and feel resigned. |
| Aasen et al. | Patient factors | Barrier | Trust and Power | Feeling controlled | The metaphors ‘prison’, ‘guardian’ and ‘race walking’ in these quotations might tell that the patients felt controlled and incapacitated by nurses who did not give them priority or did not have time to talk with them. |
| Aasen et al. | Patient factors | Barrier | Trust and Power | Feeling incapacitated | The metaphors ‘prison’, ‘guardian’ and ‘race walking’ in these quotations might tell that the patients felt controlled and incapacitated by nurses who did not give them priority or did not have time to talk with them. |
| Aasen et al. | Healthcare personell factors | Barrier | Attitude and Behaviour | No treatment or information involvment of patients | The metaphors ‘prison’, ‘guardian’ and ‘race walking’ in these quotations might tell that the patients felt controlled and incapacitated by nurses who did not give them priority or did not have time to talk with them. |
| Aasen et al. | Healthcare personell factors | Barrier | Trust and Power | Exercising power and dominance | The metaphors ‘prison’, ‘guardian’ and ‘race walking’ in these quotations might tell that the patients felt controlled and incapacitated by nurses who did not give them priority or did not have time to talk with them. |
| Aasen et al. | Decision-making interaction factors | Barrier | Trust and Power | Submissive behaviour | The patients told that they felt they had to satisfy the nurses. |
| Aasen et al. | Patient factors | Barrier | Trust and Power | Submission towards healthcare personnel | The patients told that they felt they had to satisfy the nurses. |
| Aasen et al. | Healthcare system /organisatonal factors | Barrier | Treatment Organization and Risk | High Workload | but the patients felt pity for the nurses because they had so much to do. In small units, the patients remarked that the nurses had more time for them. The dialysis units seemed to be a context that did not promote participation in a satisfying manner. |
| Aasen et al. | Patient factors | Facilitator | Treatment Organization and Risk | Satisfying information sharing | Some patients seemed to be satisfied with the information they were given, whereas others were not. |
| Aasen et al. | Patient factors | Barrier | Health and Age | Being overstrained | The findings showed that patients who received acute dialysis treatment did not always remember what had happened and that the information provided later could be accidental. |
| Aasen et al. | Healthcare system /organisatonal factors | Barrier | Treatment Organization and Risk | Acute setting | The findings showed that patients who received acute dialysis treatment did not always remember what had happened and that the information provided later could be accidental. |
| Aasen et al. | Healthcare interactioj | Facilitator | Attitude and Behaviour | Active behaviour | Some patients said that they felt they could ask questions, whereas others were more passive and said they did not know what to ask.In particular, patients with a higher level of education expressed their satisfaction with the initial information. |
| Aasen et al. | Patient factors | Facilitator | Attitude and Behaviour | Active behaviour | Some patients said that they felt they could ask questions, whereas others were more passive and said they did not know what to ask.In particular, patients with a higher level of education expressed their satisfaction with the initial information. |
| Aasen et al. | Patient factors | Facilitator | Treatment Organization and Risk | Satisfying information sharing | Some patients said that they felt they could ask questions, whereas others were more passive and said they did not know what to ask.In particular, patients with a higher level of education expressed their satisfaction with the initial information. |
| Aasen et al. | Patient factors | Barrier | Health and Age | Being overstrained | Some patients said that they felt they could ask questions, whereas others were more passive and said they did not know what to ask.In particular, patients with a higher level of education expressed their satisfaction with the initial information. |
| Aasen et al. | Patient factors | Barrier | Trust and Power | Submissive communication semantics | The interpersonal modality or the unbalanced power between the health-care team and the patients was expressed in the patients’ use of phrases such as ‘They knew’, ‘I can ask’, ‘I want’ and ‘They could’. |
| Aasen et al. | Healthcare personell factors | Barrier | Trust and Power | Asymmetric power relationship | The interpersonal modality or the unbalanced power between the health-care team and the patients was expressed in the patients’ use of phrases such as ‘They knew’, ‘I can ask’, ‘I want’ and ‘They could’. |
| Aasen et al. | Decision-making interaction factors | Barrier | Trust and Power | Asymmetric power relationship | The interpersonal modality or the unbalanced power between the health-care team and the patients was expressed in the patients’ use of phrases such as ‘They knew’, ‘I can ask’, ‘I want’ and ‘They could’. |
| Aasen et al. | Patient factors | Barrier | Knowledge and Communication | Lack of medical or treatment related knowledge | one patient (P4) commented, ‘They probably knew much more than I can ask about. I do not know what to ask about. … I knew that I have to go to the dialysis. Is there anything else?’ |
| Aasen et al. | Patient factors | Facilitator | Knowledge and Communication | Wanting to be informed or demanding more information | Another patient (P5) remarked, ‘I want more information. … Nurses do not tell me anything, other than the blood percentage. … They could talk more about the illness and how it develops’. |
| Aasen et al. | Healthcare personell factors | Barrier | Knowledge and Communication | No treatment or information involvment of patients | Another patient (P5) remarked, ‘I want more information. … Nurses do not tell me anything, other than the blood percentage. … They could talk more about the illness and how it develops’. |
| Aasen et al. | Healthcare personell factors | Barrier | Knowledge and Communication | No treatment or information involvment of patients | Another patient (P5) remarked, ‘I want more information. … Nurses do not tell me anything, other than the blood percentage. … They could talk more about the illness and how it develops’. |
| Aasen et al. | Decision-making interaction factors | Barrier | Knowledge and Communication | Knowledge/Competence asymmetry | The patients depended on the health-care team to share knowledge with them |
| Aasen et al. | Healthcare system /organisatonal factors | Barrier | Knowledge and Communication | No treatment or information involvment of patients | Several patients said they lacked information about what might happen in the future and asked the researcher about that |
| Aasen et al. | Healthcare personell factors | Barrier | Knowledge and Communication | No treatment or information involvment of patients | Several patients said they lacked information about what might happen in the future and asked the researcher about that |
| Aasen et al. | Healthcare personell factors | Barrier | Trust and Power | Exercising power and dominance | The patients talked as if the health-care team owned the knowledge and decided what the patients needed to know. |
| Aasen et al. | Decision-making interaction factors | Barrier | Knowledge and Communication | Knowledge/Competence asymmetry | The patients talked as if the health-care team owned the knowledge and decided what the patients needed to know. |
| Aasen et al. | Healthcare personell factors | Barrier | Trust and Power | No treatment or information involvment of patients | The patients talked as if the health-care team owned the knowledge and decided what the patients needed to know. |
| Aasen et al. | Patient factors | Barrier | Trust and Power | Trust towards healthcare personnel | All the patients said that they trusted the health-care team. Patients who had been undergoing dialysis for <2 years had become better of their illness and were afraid of what might happen if they refused to do as they were told. |
| Aasen et al. | Patient factors | Barrier | Trust and Power | Fear of incompliance | All the patients said that they trusted the health-care team. Patients who had been undergoing dialysis for <2 years had become better of their illness and were afraid of what might happen if they refused to do as they were told. |
| Aasen et al. | Patient factors | Barrier | Trust and Power | Asymmetric power relationship | All the patients said that they trusted the health-care team. Patients who had been undergoing dialysis for <2 years had become better of their illness and were afraid of what might happen if they refused to do as they were told. |
| Aasen et al. | Decision-making interaction factors | Barrier | Trust and Power | Asymmetric power relationship | P6: First, I have to say that when you start dialysis, you must accept what they think about your situation. You can’t say that what they say is nonsense. It’s no good to attend dialysis and then start discussing the matter. The people who treat you are professionals. These doctors always think they ought to decide and that I should listen to them. And maybe they are right because if I don’t, then it may not end up so well |
| Aasen et al. | Patient factors | Barrier | Knowledge and Communication | Knowledge/Competence asymmetry | P6: First, I have to say that when you start dialysis, you must accept what they think about your situation. You can’t say that what they say is nonsense. It’s no good to attend dialysis and then start discussing the matter. The people who treat you are professionals. These doctors always think they ought to decide and that I should listen to them. And maybe they are right because if I don’t, then it may not end up so well |
| Aasen et al. | Patient factors | Barrier | Attitude and Behaviour | Submissive behaviour | P6: First, I have to say that when you start dialysis, you must accept what they think about your situation. You can’t say that what they say is nonsense. It’s no good to attend dialysis and then start discussing the matter. The people who treat you are professionals. These doctors always think they ought to decide and that I should listen to them. And maybe they are right because if I don’t, then it may not end up so well |
| Aasen et al. | Patient factors | Barrier | Knowledge and Communication | Lack of medical or treatment related knowledge | P6: First, I have to say that when you start dialysis, you must accept what they think about your situation. You can’t say that what they say is nonsense. It’s no good to attend dialysis and then start discussing the matter. The people who treat you are professionals. These doctors always think they ought to decide and that I should listen to them. And maybe they are right because if I don’t, then it may not end up so well |
| Aasen et al. | Healthcare personell factors | Barrier | Trust and Power | Exercising power and dominance | P6: First, I have to say that when you start dialysis, you must accept what they think about your situation. You can’t say that what they say is nonsense. It’s no good to attend dialysis and then start discussing the matter. The people who treat you are professionals. These doctors always think they ought to decide and that I should listen to them. And maybe they are right because if I don’t, then it may not end up so well |
| Aasen et al. | Patient factors | Barrier | Knowledge and Communication | Lack of medical or treatment related knowledge | P6: First, I have to say that when you start dialysis, you must accept what they think about your situation. You can’t say that what they say is nonsense. It’s no good to attend dialysis and then start discussing the matter. The people who treat you are professionals. These doctors always think they ought to decide and that I should listen to them. And maybe they are right because if I don’t, then it may not end up so well |
| Aasen et al. | Patient factors | Barrier | Attitude and Behaviour | Submissive behaviour | If you begin dialysis, then you should listen to the staff who talks to you because they know what they’re doing. Even if we want to decide what should happen, it doesn’t mean that we could. You must listen to those who are supposed to help you, or else it won’t work. … I don’t know, but I started trusting the ones who were treating me. |
| Aasen et al. | Patient factors | Barrier | Trust and Power | Trust towards healthcare personnel | If you begin dialysis, then you should listen to the staff who talks to you because they know what they’re doing. Even if we want to decide what should happen, it doesn’t mean that we could. You must listen to those who are supposed to help you, or else it won’t work. … I don’t know, but I started trusting the ones who were treating me. |
| Aasen et al. | Decision-making interaction factors | Barrier | Knowledge and Communication | Knowledge/Competence asymmetry | If you begin dialysis, then you should listen to the staff who talks to you because they know what they’re doing. Even if we want to decide what should happen, it doesn’t mean that we could. You must listen to those who are supposed to help you, or else it won’t work. … I don’t know, but I started trusting the ones who were treating me. |
| Aasen et al. | Patient factors | Barrier | Trust and Power | Fear of incompliance | If you begin dialysis, then you should listen to the staff who talks to you because they know what they’re doing. Even if we want to decide what should happen, it doesn’t mean that we could. You must listen to those who are supposed to help you, or else it won’t work. … I don’t know, but I started trusting the ones who were treating me. |
| Aasen et al. | Patient factors | Barrier | Trust and Power | Fear of incompliance | This combination of trust and fear might create passiveness and be a possible obstacle to dialogue and shared decisionmaking. |
| Aasen et al. | Decision-making interaction factors | Barrier | Trust and Power | Trust towards healthcare personnel | This combination of trust and fear might create passiveness and be a possible obstacle to dialogue and shared decisionmaking. |
| Aasen et al. | Healthcare personell factors | Barrier | Trust and Power | Being ignored/Ignoring patients | The act of ignoring somebody is also a way to dominate other people. Although patients who had been undergoing dialysis for years and were in a stable phase of treatment still trusted the health-care team, they expressed some dissatisfaction |
| Aasen et al. | Healthcare personell factors | Barrier | Knowledge and Communication | No treatment or information involvment of patients | The act of ignoring somebody is also a way to dominate other people. Although patients who had been undergoing dialysis for years and were in a stable phase of treatment still trusted the health-care team, they expressed some dissatisfaction |
| Aasen et al. | Healthcare personell factors | Barrier | Trust and Power | Asymmetric power relationship | The act of ignoring somebody is also a way to dominate other people. Although patients who had been undergoing dialysis for years and were in a stable phase of treatment still trusted the health-care team, they expressed some dissatisfaction |
| Aasen et al. | Decision-making interaction factors | Barrier | Trust and Power | Asymmetric power relationship | The act of ignoring somebody is also a way to dominate other people. Although patients who had been undergoing dialysis for years and were in a stable phase of treatment still trusted the health-care team, they expressed some dissatisfaction |
| Aasen et al. | Healthcare personell factors | Barrier | Trust and Power | Being ignored/Ignoring patients | They probably have got tired of me after so many years. Probably, they aren’t that interested anymore. It’s like I’ve become a piece of furniture |
| Aasen et al. | Healthcare personell factors | Barrier | Knowledge and Communication | No treatment or information involvment of patients | They probably have got tired of me after so many years. Probably, they aren’t that interested anymore. It’s like I’ve become a piece of furniture |
| Aasen et al. | Patient factors | Barrier | Knowledge and Communication | Submissive communication semantics | These patients used metaphors like ‘furniture’ and ‘package’ and words of appraisal such as ‘isolated’ and ‘they aren’t that interested’. |
| Aasen et al. | Patient factors | Barrier | Trust and Power | Passive behaviour | Some of the patients felt that the health-care team might have forgotten about them. They missed the dialogue and felt powerless. |
| Aasen et al. | Patient factors | Barrier | Trust and Power | Feeling powerless / Having no control | Some of the patients felt that the health-care team might have forgotten about them. They missed the dialogue and felt powerless. |
| Aasen et al. | Patient factors | Facilitator | Attitude and Behaviour | Wanting to be involved through decisional participation | Some of the patients felt that the health-care team might have forgotten about them. They missed the dialogue and felt powerless. |
| Aasen et al. | Healthcare personell factors | Barrier | Knowledge and Communication | Dominant communication semantics | The patients’ identities seemed to be influenced by this situation, and they found it difficult to be active. |
| Aasen et al. | Patient factors | Facilitator | Attitude and Behaviour | Active behaviour | Several patients said that they preferred the catheter, but there was no real choice because the health-care team preferred the fistula. Patients were told that the fistula was the better option because its use was associated with fewer infections and better dialysis. However, from the patients’ perspective, there were questions regarding problems with the fistula |
| Aasen et al. | Patient factors | Facilitator | Knowledge and Communication | Wanting to express themselves, issue opinions and preferences and to be heard | Several patients said that they preferred the catheter, but there was no real choice because the health-care team preferred the fistula. Patients were told that the fistula was the better option because its use was associated with fewer infections and better dialysis. However, from the patients’ perspective, there were questions regarding problems with the fistula |
| Aasen et al. | Decision-making interaction factors | Barrier | Trust and Power | Asymmetric power relationship | ‘‘‘It will be what I say it will be’’, she [a member of the health-care team] says. ‘‘Yes, that’s OK,’’ I say, ‘‘but I should be allowed to protest’’’. |
| Aasen et al. | Healthcare personell factors | Barrier | Trust and Power | Exercising power and dominance | ‘‘‘It will be what I say it will be’’, she [a member of the health-care team] says. ‘‘Yes, that’s OK,’’ I say, ‘‘but I should be allowed to protest’’’. |
| Aasen et al. | Healthcare personell factors | Barrier | Trust and Power | Being ignored/Ignoring patients | The patients did not always agree and tried to argue with the health-care team that they, for example, had a higher natural weight. Their well-being (during and after dialysis) was connected to how much water the treatment removed. The patients wanted to have something to say about their dry weight, but it was difficult to obtain their opinions across. |
| Aasen et al. | Decision-making interaction factors | Barrier | Trust and Power | Asymmetric power relationship | The patients did not always agree and tried to argue with the health-care team that they, for example, had a higher natural weight. Their well-being (during and after dialysis) was connected to how much water the treatment removed. The patients wanted to have something to say about their dry weight, but it was difficult to obtain their opinions across. |
| Aasen et al. | Patient factors | Facilitator | Knowledge and Communication | Wanting to express themselves, issue opinions and preferences and to be heard | For several patients, maintaining a good quality of lifetoday was more important than having a ‘long’ life. Three patients admitted that they did not comply with the diet.The health-care team explained the consequences of not following the diet, but these patients did not feel any worse if they did not comply. Therefore, they wielded their power and did not comply with the diet. |
| Aasen et al. | Healthcare system /organisatonal factors | Barrier | Treatment Organization and Risk | Lack of integration in social practices | The patients perceived a social practice in the dialysis units where the health-care teams have the power and dominance |
| Aasen et al. | Decision-making interaction factors | Barrier | Trust and Power | Asymmetric power relationship | The patients perceived a social practice in the dialysis units where the health-care teams have the power and dominance |
| Aasen et al. | Healthcare personell factors | Barrier | Trust and Power | Exercising power and dominance | The patients perceived a social practice in the dialysis units where the health-care teams have the power and dominance |
| Aasen et al. | Decision-making interaction factors | Barrier | Trust and Power | Asymmetric power relationship | the order of the discourse and the ideology in the patients’ stories were mostly paternalistic, and the patients played minimal and passive roles. |
| Aasen et al. | Patient factors | Barrier | Trust and Power | Feeling controlled | The conditions for participation are influenced by the context (23, 39, 42, 43). Similar to the findings of earlier studies (35, 36), the patients in the present study described a context in which they were controlled, sensed a loss of freedom and felt powerless. |
| Aasen et al. | Patient factors | Barrier | Trust and Power | Feeling powerless / Having no control | The conditions for participation are influenced by the context (23, 39, 42, 43). Similar to the findings of earlier studies (35, 36), the patients in the present study described a context in which they were controlled, sensed a loss of freedom and felt powerless. |
| Aasen et al. | Patient factors | Barrier | Trust and Power | Feeling incapacitated | The conditions for participation are influenced by the context (23, 39, 42, 43). Similar to the findings of earlier studies (35, 36), the patients in the present study described a context in which they were controlled, sensed a loss of freedom and felt powerless. |
| Aasen et al. | Healthcare system /organisatonal factors | Barrier | Treatment Organization and Risk | High Workload | The health-care team was often in a hurry and was not available for engaging in dialogue and in making negotiations. |
| Aasen et al. | Healthcare system /organisatonal factors | Facilitator | Treatment Organization and Risk | Adequate workload | The organisation and structure of the dialysis units seem to be important for participation. Patients from smaller units with few patients and those from larger units visiting smaller units spoke of an obliging atmosphere where nurses had time for them. |
| Aasen et al. | Healthcare system /organisatonal factors | Barrier | Treatment Organization and Risk | Lack of integration in social practices | The context of the dialysis units did not seem to promote patient participation. Changing the social practice in dialysis units from a paternalistic ideology to an ideology of participation will require the health-care team to engage in the following activities |
| Bleicher et al. | Patient factors | Facilitator | Attitude and Behaviour | Active behaviour | Older women participate equally in breast cancer surgical decision making and are equally likely to select mastectomy, but use less knowledge to make the decision. |
| Bleicher et al. | Patient factors | Facilitator | Knowledge and Communication | Medical knowledge is not required | Older women participate equally in breast cancer surgical decision making and are equally likely to select mastectomy, but use less knowledge to make the decision. |
| Bleicher et al. | Patient factors | Facilitator | Attitude and Behaviour | Active behaviour | Overall, decisions were surgeon-based, shared, and patient-based in 23.5%, 38.1%, and 38.4% of cases, respectively, |
| Bleicher et al. | Patient factors | Facilitator | Treatment Organization and Risk | Satisfying involvment | Between 48% and 57% of the women in each age group felt that they were able to participate in the decision to the extent that they desired |
| Bleicher et al. | Patient factors | Barrier | Health and Age | Discomfort due to too much involvement | This may be a function of patients ‘‘shopping around’’ for a recommendation that supports their preconceived view that mastectomy is better or may indicate discomfort with being more involved in the decision-making process than they desired. |
| Bleicher et al. | Patient factors | Facilitator | Attitude and Behaviour | Confidence in participating in decisional involvment | As illustrated in Fig. 3, no significant differences in patient confidence in decision making were seen between the age groups (p = 0.23), with the overwhelming majority of each group feeling either extremely or very confident about their ability to participate in the decision-making process. |
| Bleicher et al. | Patient factors | Facilitator | Knowledge and Communication | Wanting to express themselves, issue opinions and preferences and to be heard | Patients were also surveyed about concerns contributing to their treatment choice. Most of the responses were not age-related. These included concerns about convenience, affordability, long-term surgical or radiotherapy side effects, the need for a second surgical procedure, a quicker recovery, less pain, the ability to wear regular clothes, radiation exposure, and resumption of regular activities |
| Bleicher et al. | Patient factors | Facilitator | Attitude and Behaviour | Wanting to be involved through decisional participation | This study found that only a small minority of women would have preferred a greater role in surgical decision making than they had, and this was independent of age |
| Bleicher et al. | Patient factors | Facilitator | Knowledge and Communication | Medical knowledge is not required | Some aspects of decision making did vary with age. The oldest women responded ‘‘I dont know’’ more frequently to knowledge questions than their younger counterparts, |
| Bleicher et al. | Patient factors | Facilitator | Knowledge and Communication | Medical knowledge is not required | In our study, older age was associated with a significantly higher percentage of ‘‘I dont know’’ responses, but no diminution of confidence in the ability to participate in decision making. This suggests that older women make their decisions with less factual knowledge about their choice, |
| Bleicher et al. | Patient factors | Facilitator | Attitude and Behaviour | Confidence in participating in decisional involvment | In our study, older age was associated with a significantly higher percentage of ‘‘I dont know’’ responses, but no diminution of confidence in the ability to participate in decision making. This suggests that older women make their decisions with less factual knowledge about their choice, |
| Bleicher et al. | Patient factors | Facilitator | Attitude and Behaviour | Active behaviour | The number of surgeons consulted preoperatively did not vary significantly by age, although consultation with a greater number of surgeons did predict mastectomy choice. This may be a function of patients ‘‘shopping around’’ for a recommendation that supports their preconceived view |
| Dardas et al. | Patient factors | Facilitator | Attitude and Behaviour | Active behaviour | Eighty one percent of patients analyzed preferred a more patient-directed role in decision making, with 46% of the total cohort citing a collaborative approach as their most preferred treatment approach |
| Dardas et al. | Patient factors | Facilitator | Attitude and Behaviour | Wanting to be involved through decisional participation | Eighty one percent of patients analyzed preferred a more patient-directed role in decision making, with 46% of the total cohort citing a collaborative approach as their most preferred treatment approach |
| Dardas et al. | Patient factors | Facilitator | Knowledge and Communication | Wanting to be informed or demanding more information | Forty-nine percent reported that spending more time with their physician to address questions and explain the diagnosis would be most useful when making a health care decision and 73% preferred additional written informational material |
| Dardas et al. | Patient factors | Facilitator | Attitude and Behaviour | Wanting to be involved through decisional participation | Forty-nine percent reported that spending more time with their physician to address questions and explain the diagnosis would be most useful when making a health care decision and 73% preferred additional written informational material |
| Dardas et al. | Patient factors | Facilitator | Attitude and Behaviour | Wanting to be involved through decisional participation | Older adult patients with symptomatic upper extremity conditions desire more patient-directed roles in treatment decision making |
| Dardas et al. | Patient factors | Facilitator | Attitude and Behaviour | Wanting to be involved through decisional participation | Eighty-one percent of patients stated they preferred a more active role in decision-making. |
| Dardas et al. | Patient factors | Barrier | Knowledge and Communication | Depending on family or healthcare personnel | Forty percent relied on family to help with decisions and 38% relied on their primary care provider |
| Dardas et al. | Patient factors | Facilitator | Knowledge and Communication | Wanting to be informed or demanding more information | Spending more time with a physician addressing questions and explaining the diagnosis was most frequently ranked as useful to making a healthcare decision (49%). |
| Dardas et al. | Patient factors | Facilitator | Attitude and Behaviour | Active behaviour | The top 3 sources of background medical information for this population were information from their physician during a clinic appointment (69%), internet searches for credible medical websites (35%), and internet searches for any relevant information without attention to source (18%). |
| Dardas et al. | Patient factors | Facilitator | Knowledge and Communication | Internet as source for medical information | The top 3 sources of background medical information for this population were information from their physician during a clinic appointment (69%), internet searches for credible medical websites (35%), and internet searches for any relevant information without attention to source (18%). |
| Dardas et al. | Patient factors | Facilitator | Knowledge and Communication | Wanting to be informed or demanding more information | Sixty-two percent indicated that more information about their diagnosis prior to an appointment would improve the usefulness of the appointment |
| Dardas et al. | Patient factors | Facilitator | Attitude and Behaviour | Wanting to be involved through decisional participation | The most frequently preferred card on the Control Preferences Scale was C, which illustrated equitable decision-making with shared responsibility between the patient and doctor, chosen by 46 participants (46%). |
| Dardas et al. | Patient factors | Facilitator | Attitude and Behaviour | Wanting to be involved through decisional participation | Card B, representing patient-directed collaboration, was second most preferred, chosen by 27 participants (27%), followed by card A, the most patient-directed end of the scale by 14 subjects (14%). Combined, 87% of patients preferred to be in the shared to patient-directed spectrum of the scale, indicating they wanted to have at least an equal, if not dominant, rolein their ideal health care visit |
| Dardas et al. | Patient factors | Facilitator | Knowledge and Communication | Medical knowledge is not required | There were no associations between patient age, sex, education level, working status, living arrangement, health literacy measures, patient-reported health status, or magnitude of upper extremity disability with the preferred role in treatment decision-making |
| Dardas et al. | Patient factors | Facilitator | Trust and Power | Unknown healthcare provider | Multi-nomial logistic regression revealed that being new to a provider significantly predicted patient preference for a patient-directed role in decision making against a reference standard of the physician-directed role (P<0.05, ExpB 6.2, 95% CI 1.1–36.0) even when accounting for health literacy (P=0.90) and PRWE score (P=0.46). |
| Dardas et al. | Patient factors | Facilitator | Attitude and Behaviour | Wanting to be involved through decisional participation | While age continues to impact decision-making preferences, 82% of patients over 60 years old want to share or direct their medical decisions when discussing advanced care planning.2 Our data similarly indicate that when receiving elective specialty hand surgical care, older adult patients want to remain actively involved in their treatment decisions and use their physician visit as their primary source of information. |
| Dardas et al. | Patient factors | Facilitator | Knowledge and Communication | Wanting to be informed or demanding more information | While age continues to impact decision-making preferences, 82% of patients over 60 years old want to share or direct their medical decisions when discussing advanced care planning.2 Our data similarly indicate that when receiving elective specialty hand surgical care, older adult patients want to remain actively involved in their treatment decisions and use their physician visit as their primary source of information. |
| Dardas et al. | Healthcare personell factors | Barrier | Treatment Organization and Risk | Time pressure | The conflict between limited physician time and the need for “preference-sensitive care” requires tools that help assess patient goals and provide unbiased information without creating an undue time burden on providers in clinic. |
| Dardas et al. | Healthcare personell factors | Facilitator | Treatment Organization and Risk | High Workload | The conflict between limited physician time and the need for “preference-sensitive care” requires tools that help assess patient goals and provide unbiased information without creating an undue time burden on providers in clinic. |
| Dardas et al. | Patient factors | Facilitator | Knowledge and Communication | Wanting to be informed or demanding more information | Validated patient decision aids are powerful tools to educate patients about the risks and benefits regarding available treatment options and engage them in discussing personal preferences and beliefs about their care and condition.26 In doing so, patient knowledge and satisfaction improves and patient decision conflict diminishes. |
| Dardas et al. | Patient factors | Facilitator | Treatment Organization and Risk | Treatment satisfaction | In doing so, patient knowledge and satisfaction improves and patient decision conflict diminishes |
| Dardas et al. | Patient factors | Facilitator | Treatment Organization and Risk | Diminution of decisional conflict | In doing so, patient knowledge and satisfaction improves and patient decision conflict diminishes |
| Dardas et al. | Patient factors | Facilitator | Trust and Power | Unknown healthcare provider | Our data demonstrate that familiarity with the physician may be a modifier of patient preferences in decision making as return patients were more likely to prefer a shared approach and new patients were more likely to prefer a patient-directed role. This may reflect increased patient trust in a provider’s understanding of their goals and values and willingness to share a decision |
| Deme et al. | Patient factors | Barrier | Attitude and Behaviour | Not having a choice | patients felt surgery was their only choice because they were running out of time to undergo invasive procedures |
| Deme et al. | Patient factors | Barrier | Treatment Organization and Risk | Timely treatment necessity | patients felt surgery was their only choice because they were running out of time to undergo invasive procedures |
| Deme et al. | Patient factors | Facilitator | Attitude and Behaviour | Active behaviour | patients mentally committed to surgery prior to the initial encounter with their surgeon and contextualized the desired benefits while minimizing the potential risks |
| Deme et al. | Patient factors | Barrier | Attitude and Behaviour | Not having a choice | patients mentally committed to surgery prior to the initial encounter with their surgeon and contextualized the desired benefits while minimizing the potential risks |
| Deme et al. | Healthcare personell factors | Barrier | Attitude and Behaviour | Diverging understanding and implementation of SDM | surgeons varied substantially in their interpretations of shared decision making |
| Deme et al. | Decision-making interaction factors | Barrier | Knowledge and Communication | Diverging perceptions of health condition, treatment or surgical outcome | there is a goal mismatch between patients and surgeons in the desired outcomes from surgery, where patients prioritize complete pain relief whereas surgeons prioritize concrete functional improvement |
| Deme et al. | Decision-making interaction factors | Barrier | Attitude and Behaviour | Diverging understanding and implementation of SDM | These findings highlight the need for improved understanding of both sides of shared decision making which should involve the needs and priorities of older adults to help convey patient-specific risks and choice awareness |
| Deme et al. | Patient factors | Barrier | Attitude and Behaviour | Not having a choice | Running out of time: Patients felt that their only choice was to undergo spinal deformity correction because they were running out of time to undergo invasive procedure given their age |
| Deme et al. | Patient factors | Barrier | Health and Age | Timely treatment necessity | Running out of time: Patients felt that their only choice was to undergo spinal deformity correction because they were running out of time to undergo invasive procedure given their age |
| Deme et al. | Patient factors | Barrier | Attitude and Behaviour | Not having a choice | …In my case, the consideration was the fact that there was a window during which I could have this surgery and if I passed that window nobody was going to want to do it. |
| Deme et al. | Patient factors | Barrier | Treatment Organization and Risk | Timely treatment necessity | …In my case, the consideration was the fact that there was a window during which I could have this surgery and if I passed that window nobody was going to want to do it. |
| Deme et al. | Patient factors | Barrier | Attitude and Behaviour | Not having a choice | Pain was the primary motivator for pursuing surgery in all patients. Some patients addressed functional status, but still identified pain as the principal driver for seeking care. …I just wanted anything that would give me relief. I was desperate |
| Deme et al. | Patient factors | Facilitator | Attitude and Behaviour | Active behaviour | Pre-conceived decision-making: Patients mentally committed to surgery prior to the initial encounter with their surgeon and contextualized the desired benefits of surgery while minimizing the potential risks. |
| Deme et al. | Patient factors | Barrier | Attitude and Behaviour | Not having a choice | …I made my mind up I was going to have surgery before we(my surgeon and I) even talkedabout it. |
| Deme et al. | Patient factors | Barrier | Attitude and Behaviour | Not having a choice | I really didn't think that much about the risks because I wanted the surgery. But I guess, you know, there was a small chance that a complication could happen. But that would not have stopped me. |
| Deme et al. | Patient factors | Facilitator | Attitude and Behaviour | Active behaviour | I really didn't think that much about the risks because I wanted the surgery. But I guess, you know, there was a small chance that a complication could happen. But that would not have stopped me. |
| Deme et al. | Patient factors | Facilitator | Knowledge and Communication | Wanting to express themselves, issue opinions and preferences and to be heard | …I made my mind up I was going to have surgery before we(my surgeon and I) even talked about it. |
| Deme et al. | Patient factors | Facilitator | Trust and Power | Exercising power and dominance | I really didn't think that much about the risks because I wanted the surgery. But I guess, you know, there was a small chance that a complication could happen. But that would not have stopped me. |
| Deme et al. | Patient factors | Facilitator | Trust and Power | Exercising power and dominance | …I made my mind up I was going to have surgery before we(my surgeon and I) even talked about it. |
| Deme et al. | Patient factors | Facilitator | Attitude and Behaviour | Confidence in participating in decisional involvment | I really didn't think that much about the risks because I wanted the surgery. But I guess, you know, there was a small chance that a complication could happen. But that would not have stopped me. |
| Deme et al. | Patient factors | Facilitator | Attitude and Behaviour | Confidence in participating in decisional involvment | …I made my mind up I was going to have surgery before we(my surgeon and I) even talked about it. |
| Deme et al. | Patient factors | Facilitator | Attitude and Behaviour | Wanting to be involved through decisional participation | I really didn't think that much about the risks because I wanted the surgery. But I guess, you know, there was a small chance that a complication could happen. But that would not have stopped me. |
| Deme et al. | Patient factors | Facilitator | Attitude and Behaviour | Wanting to be involved through decisional participation | …I made my mind up I was going to have surgery before we(my surgeon and I) even talked about it. |
| Deme et al. | Patient factors | Facilitator | Knowledge and Communication | Wanting to be informed or demanding more information | wish I knew more about the surgery. The in-person classes, brochures and videos were not effective in helping me understand what surgery entailed. |
| Deme et al. | Patient factors | Barrier | Knowledge and Communication | No treatment or information involvment of patients | wish I knew more about the surgery. The in-person classes, brochures and videos were not effective in helping me understand what surgery entailed. |
| Deme et al. | Healthcare personell factors | Barrier | Attitude and Behaviour | Diverging understanding and implementation of SDM | Interpretations of SDM: While spine surgeons intuitively understood the concept of shared decision-making, they varied substantially in their interpretations. All surgeons understood that SDM is a framework to involve the patient, patient’s support system, and provider in medical decisionmaking. |
| Deme et al. | Healthcare personell factors | Barrier | Attitude and Behaviour | Diverging understanding and implementation of SDM | The majority of surgeons described family involvement within SDM. Some interpreted this as a separate definition of shared decision-making. …Alternatively, there could be shared decision-making on the part of the family or those on the patient side making decisions, so I think there's a couple different ways to interpret that. |
| Deme et al. | Decision-making interaction factors | Barrier | Knowledge and Communication | Diverging perceptions of health condition, treatment or surgical outcome | Goal Mismatch: There is a goal mismatch between patients and surgeons in the desired outcomes from surgery where patients prioritize complete pain relief whereas surgeons prioritize concrete functional improvement. |
| Deme et al. | Decision-making interaction factors | Barrier | Knowledge and Communication | Diverging perceptions of health condition, treatment or surgical outcome | if [surgery] doesn't take away all of their pain, but hopefully moderately improves it such that they(patients) can be more active, then we would have accomplished the goals of surgery. |
| Deme et al. | Decision-making interaction factors | Barrier | Knowledge and Communication | Diverging perceptions of health condition, treatment or surgical outcome | However, many patients present with pain and value pain relief as benchmark of satisfaction after surgery. This can be a large driver of regret. …Yes, persistent pain is the primary reason for decisional regret. If patients do not have complete pain relief after surgery, they often question the success of the surgery |
| Deme et al. | Patient factors | Barrier | Health and Age | Forgetting discussions or given information | Older adults undergoing spinal deformity surgery were shown to have poor recall with only 45% recall immediately after discussion and 18% at 6 weeks post-operatively, suggesting an important role in ensuring repetitive emphasis on surgical risk and benefits |
| Deme et al. | Patient factors | Barrier | Knowledge and Communication | Depending on family or healthcare personnel | Family support was identified as a critical component of success in the post-operative course, and naturally, family should be included in the decision-making process. This is strengthened by the fact that recall is improved in older patients when family is present during discussion, from 45% to 55% immediately after and 36% from 18% at 6 to 8 weeks after surgery15 |
| Deme et al. | Patient factors | Barrier | Health and Age | Forgetting discussions or given information | Family support was identified as a critical component of success in the post-operative course, and naturally, family should be included in the decision-making process. This is strengthened by the fact that recall is improved in older patients when family is present during discussion, from 45% to 55% immediately after and 36% from 18% at 6 to 8 weeks after surgery15 |
| Deme et al. | Patient factors | Barrier | Health and Age | Timely treatment necessity | However, patients focused on chronological age and felt pressured to have surgery due to “limited time”. |
| Deme et al. | Decision-making interaction factors | Barrier | Knowledge and Communication | Diverging perceptions of health condition, treatment or surgical outcome | Similarly, there was notable misalignment in defining success of surgery. Surgeons focus on relative improvement in functionality, whereas patients may perseverate on remaining pain. |
| Deme et al. | Decision-making interaction factors | Barrier | Knowledge and Communication | Diverging perceptions of health condition, treatment or surgical outcome | There is a goal mismatch between patients and surgeons in the desired outcomes from surgery, where patients prioritize complete pain relief whereas surgeons prioritize concrete functional improvement |
| Ekdahl et al. | Patient factors | Facilitator | Knowledge and Communication | Wanting to be informed or demanding more information | Patient participation to frail elderly means information, not the wish to take part in decisions about their medical treatments |
| Ekdahl et al. | Patient factors | Barrier | Attitude and Behaviour | Not wanting to participate in decision making | Patient participation to frail elderly means information, not the wish to take part in decisions about their medical treatments |
| Ekdahl et al. | Patient factors | Barrier | Trust and Power | Institution of power and/or trust | They view the hospital care system as an institution of power with which they cannot argue. |
| Ekdahl et al. | Patient factors | Barrier | Trust and Power | Submissive behaviour | They view the hospital care system as an institution of power with which they cannot argue. |
| Ekdahl et al. | Healthcare system /organisatonal factors | Barrier | Treatment Organization and Risk | Healthcare staff rotation | Participation is complicated by barriers such as the numerous persons involved in their care who do not know them and their preferences, differing treatment strategies among doctors, fast patient turnover in hospitals, stressed personnel and linguistic problems due to doctors not always speaking the patient’s own language. |
| Ekdahl et al. | Healthcare system /organisatonal factors | Barrier | Treatment Organization and Risk | Patient turnover | Participation is complicated by barriers such as the numerous persons involved in their care who do not know them and their preferences, differing treatment strategies among doctors, fast patient turnover in hospitals, stressed personnel and linguistic problems due to doctors not always speaking the patient’s own language. |
| Ekdahl et al. | Healthcare system /organisatonal factors | Barrier | Treatment Organization and Risk | High Workload | Participation is complicated by barriers such as the numerous persons involved in their care who do not know them and their preferences, differing treatment strategies among doctors, fast patient turnover in hospitals, stressed personnel and linguistic problems due to doctors not always speaking the patient’s own language. |
| Ekdahl et al. | Decision-making interaction factors | Barrier | Knowledge and Communication | Linguistic issues | Participation is complicated by barriers such as the numerous persons involved in their care who do not know them and their preferences, differing treatment strategies among doctors, fast patient turnover in hospitals, stressed personnel and linguistic problems due to doctors not always speaking the patient’s own language. |
| Ekdahl et al. | Healthcare personell factors | Barrier | Knowledge and Communication | Linguistic issues | Participation is complicated by barriers such as the numerous persons involved in their care who do not know them and their preferences, differing treatment strategies among doctors, fast patient turnover in hospitals, stressed personnel and linguistic problems due to doctors not always speaking the patient’s own language. |
| Ekdahl et al. | Patient factors | Facilitator | Knowledge and Communication | Wanting to be informed or demanding more information | The results of the study show that, to frail elderly patients, participation inmedical decision making is primarily a question of good communication and information, not participation in decisions about medical treatments. |
| Ekdahl et al. | Patient factors | Facilitator | Attitude and Behaviour | Not wanting to participate in decision making | The results of the study show that, to frail elderly patients, participation inmedical decision making is primarily a question of good communication and information, not participation in decisions about medical treatments. |
| Ekdahl et al. | Healthcare personell factors | Barrier | Knowledge and Communication | Comprehensive communication | The results of the study show that, to frail elderly patients, participation inmedical decision making is primarily a question of good communication and information, not participation in decisions about medical treatments. |
| Ekdahl et al. | Patient factors | Facilitator | Treatment Organization and Risk | Satisfying involvment | The results of the study show that, to frail elderly patients, participation inmedical decision making is primarily a question of good communication and information, not participation in decisions about medical treatments. |
| Ekdahl et al. | Healthcare system /organisatonal factors | Barrier | Treatment Organization and Risk | Healthcare staff rotation | More time should be given to thorough information and as few people as possibleshould be involved in the care of frail elderly |
| Ekdahl et al. | Patient factors | Facilitator | Attitude and Behaviour | Wanting to be involved through decisional participation | To the patients in this study, patient participation in medical decisions meant the receiving of information and good communication. They wanted information about their illnesses and planned investigations, and they wanted information on the results of their investigations and the doctors’ diagnostic considerations. They wanted health care staff to take the time to sit down and explain what was going to happen with them. |
| Ekdahl et al. | Patient factors | Facilitator | Knowledge and Communication | Wanting to be informed or demanding more information | To the patients in this study, patient participation in medical decisions meant the receiving of information and good communication. They wanted information about their illnesses and planned investigations, and they wanted information on the results of their investigations and the doctors’ diagnostic considerations. They wanted health care staff to take the time to sit down and explain what was going to happen with them. |
| Ekdahl et al. | Patient factors | Facilitator | Attitude and Behaviour | Wanting to be involved through decisional participation | Further, they wanted to be given the chance to be heard and to have the opportunity to express their thoughts and feelings about their symptoms and illnesses. It was important to be informed, even for those who did not wish to take an active part in the communication themselves |
| Ekdahl et al. | Patient factors | Facilitator | Knowledge and Communication | Wanting to express themselves, issue opinions and preferences and to be heard | Further, they wanted to be given the chance to be heard and to have the opportunity to express their thoughts and feelings about their symptoms and illnesses. It was important to be informed, even for those who did not wish to take an active part in the communication themselves |
| Ekdahl et al. | Patient factors | Facilitator | Knowledge and Communication | Wanting to be informed or demanding more information | Further, they wanted to be given the chance to be heard and to have the opportunity to express their thoughts and feelings about their symptoms and illnesses. It was important to be informed, even for those who did not wish to take an active part in the communication themselves |
| Ekdahl et al. | Patient factors | Barrier | Health and Age | Being ill | One problem was patients’ being too ill, for example, being too tired, being in pain or being too confused to communicate or to be interested in doing so and, thereby, to participate in their care. |
| Ekdahl et al. | Patient factors | Barrier | Health and Age | Being tired | One problem was patients’ being too ill, for example, being too tired, being in pain or being too confused to communicate or to be interested in doing so and, thereby, to participate in their care. |
| Ekdahl et al. | Patient factors | Barrier | Health and Age | Being in pain | One problem was patients’ being too ill, for example, being too tired, being in pain or being too confused to communicate or to be interested in doing so and, thereby, to participate in their care. |
| Ekdahl et al. | Patient factors | Barrier | Health and Age | Being confused | One problem was patients’ being too ill, for example, being too tired, being in pain or being too confused to communicate or to be interested in doing so and, thereby, to participate in their care. |
| Ekdahl et al. | Patient factors | Barrier | Health and Age | Being old | Due to advanced age and illnesses, the patients had extra difficulties understanding information [31] — something they were often well aware of. Many chose not to care so much about information and expressed that they were not as interested to know as they were when they were younger. |
| Ekdahl et al. | Patient factors | Barrier | Health and Age | Being overstrained | Well — that’s obvious. When you get into hospital it can be a bit stressed, and you can get a bit confused. One is not totally perfect. |
| Ekdahl et al. | Patient factors | Barrier | Attitude and Behaviour | Passive behaviour | Pat: No, I am not really taking part in my care. Int: But do you want to? Pat: No. Not now, when I am this old. Perhaps when I was younger, but not now. |
| Ekdahl et al. | Patient factors | Barrier | Health and Age | Being old | Pat: No, I am not really taking part in my care. Int: But do you want to? Pat: No. Not now, when I am this old. Perhaps when I was younger, but not now. |
| Ekdahl et al. | Healthcare system /organisatonal factors | Barrier | Treatment Organization and Risk | Healthcare staff rotation | Patients also had trouble dealing with continually changing doctors. New doctors appeared practically every day, sometimes with different treatment strategies. This made it difficult for the patient to feel confident in treatment strategies and to follow the thoughts and plans made by the doctors. |
| Ekdahl et al. | Patient factors | Barrier | Treatment Organization and Risk | Facing diverging treatment strategies | Patients also had trouble dealing with continually changing doctors. New doctors appeared practically every day, sometimes with different treatment strategies. This made it difficult for the patient to feel confident in treatment strategies and to follow the thoughts and plans made by the doctors. |
| Ekdahl et al. | Healthcare personell factors | Barrier | Treatment Organization and Risk | Facing diverging treatment strategies | Patients also had trouble dealing with continually changing doctors. New doctors appeared practically every day, sometimes with different treatment strategies. This made it difficult for the patient to feel confident in treatment strategies and to follow the thoughts and plans made by the doctors. |
| Ekdahl et al. | Patient factors | Barrier | Health and Age | Being confused | Patients also had trouble dealing with continually changing doctors. New doctors appeared practically every day, sometimes with different treatment strategies. This made it difficult for the patient to feel confident in treatment strategies and to follow the thoughts and plans made by the doctors. |
| Ekdahl et al. | Healthcare system /organisatonal factors | Barrier | Treatment Organization and Risk | Healthcare staff rotation | Then I was hospitalized, and they made some tests. And then at first a doctor came. She said that I should have warfarin. And then in the afternoon a male doctor came and said that he had heard about the plans to give me warfarin, but we should ignore that, because warfarin is dangerous stuff. And palpitations are a problem in 80% of all people above 80, he said. So I had to go home again. |
| Ekdahl et al. | Healthcare personell factors | Barrier | Treatment Organization and Risk | Facing diverging treatment strategies | Then I was hospitalized, and they made some tests. And then at first a doctor came. She said that I should have warfarin. And then in the afternoon a male doctor came and said that he had heard about the plans to give me warfarin, but we should ignore that, because warfarin is dangerous stuff. And palpitations are a problem in 80% of all people above 80, he said. So I had to go home again. |
| Ekdahl et al. | Patient factors | Barrier | Trust and Power | Submissive behaviour | The patients gave examples of participating less when they felt the staff were stressed and when they experienced short stays in hospital, which gave little time for information to be communicated. For example, patients did not think they had the right to oppose a decision about discharge. |
| Ekdahl et al. | Healthcare personell factors | Barrier | Treatment Organization and Risk | Time pressure | The patients gave examples of participating less when they felt the staff were stressed and when they experienced short stays in hospital, which gave little time for information to be communicated. For example, patients did not think they had the right to oppose a decision about discharge. |
| Ekdahl et al. | Healthcare system /organisatonal factors | Barrier | Treatment Organization and Risk | High Workload | The patients gave examples of participating less when they felt the staff were stressed and when they experienced short stays in hospital, which gave little time for information to be communicated. For example, patients did not think they had the right to oppose a decision about discharge. |
| Ekdahl et al. | Healthcare personell factors | Barrier | Knowledge and Communication | No treatment or information involvment of patients | The patients gave examples of participating less when they felt the staff were stressed and when they experienced short stays in hospital, which gave little time for information to be communicated. For example, patients did not think they had the right to oppose a decision about discharge. |
| Ekdahl et al. | Healthcare personell factors | Barrier | Treatment Organization and Risk | Time pressure | Int: Did you participate in the decision about your discharge? Pat: No. The doctor came. The doctor made his rounds, but I could not hear what he was saying — he was a foreigner — but that they could do no more. I had all the necessary medicines, he said. (Pat no. 9, female, 81 years) Int: Please describe when you were hospitalized some time ago — the shorts hospitalizations. Did you participate in the planning of your medical care then? Pat: No, I did not participate then. They just wanted me to be discharged as fast as possible. Int: So you did not participate in the planning of your medical care then? Pat: No — no, I did not. . . But the staff — they try. Int: Was it the doctors that were not communicating? Pat: Yes — but they had so much to do. A lot of patients to take care of. (Pat no. 2, female, 81 years) |
| Ekdahl et al. | Healthcare system /organisatonal factors | Barrier | Treatment Organization and Risk | High Workload | Int: Did you participate in the decision about your discharge? Pat: No. The doctor came. The doctor made his rounds, but I could not hear what he was saying — he was a foreigner — but that they could do no more. I had all the necessary medicines, he said. (Pat no. 9, female, 81 years) Int: Please describe when you were hospitalized some time ago — the shorts hospitalizations. Did you participate in the planning of your medical care then? Pat: No, I did not participate then. They just wanted me to be discharged as fast as possible. Int: So you did not participate in the planning of your medical care then? Pat: No — no, I did not. . . But the staff — they try. Int: Was it the doctors that were not communicating? Pat: Yes — but they had so much to do. A lot of patients to take care of. (Pat no. 2, female, 81 years) |
| Ekdahl et al. | Healthcare personell factors | Barrier | Knowledge and Communication | No treatment or information involvment of patients | Int: Did you participate in the decision about your discharge? Pat: No. The doctor came. The doctor made his rounds, but I could not hear what he was saying — he was a foreigner — but that they could do no more. I had all the necessary medicines, he said. (Pat no. 9, female, 81 years) Int: Please describe when you were hospitalized some time ago — the shorts hospitalizations. Did you participate in the planning of your medical care then? Pat: No, I did not participate then. They just wanted me to be discharged as fast as possible. Int: So you did not participate in the planning of your medical care then? Pat: No — no, I did not. . . But the staff — they try. Int: Was it the doctors that were not communicating? Pat: Yes — but they had so much to do. A lot of patients to take care of. (Pat no. 2, female, 81 years) |
| Ekdahl et al. | Healthcare personell factors | Barrier | Knowledge and Communication | Linguistic issues | In spite of good medical knowledge and confidence in the doctors several patients mentioned the problem of doctors speaking Swedish with an accent that made communication difficult and thus made participation difficult to achieve |
| Ekdahl et al. | Healthcare personell factors | Barrier | Knowledge and Communication | Linguistic issues | Well, it is difficult. Foreigners have difficulties. It is one thing— they come here and don’t speak the language. Perhaps they can know everything about patients but they have trouble in expressingthemselves. They have a different culture. That’s a problem, I think. |
| Ekdahl et al. | Patient factors | Facilitator | Treatment Organization and Risk | Satisfying information sharing | felt satisfied with information and participation.  This group expressed that they were pleased with the information they received which enabled them to participate to the degree they wanted, and they took the responsibility themselves, as patients, to ask questions, if they wanted to know more. |
| Ekdahl et al. | Patient factors | Facilitator | Attitude and Behaviour | Being enabled to ask questions and make decisions | felt satisfied with information and participation.  This group expressed that they were pleased with the information they received which enabled them to participate to the degree they wanted, and they took the responsibility themselves, as patients, to ask questions, if they wanted to know more. |
| Ekdahl et al. | Patient factors | Facilitator | Attitude and Behaviour | Active behaviour | felt satisfied with information and participation.  This group expressed that they were pleased with the information they received which enabled them to participate to the degree they wanted, and they took the responsibility themselves, as patients, to ask questions, if they wanted to know more. |
| Ekdahl et al. | Patient factors | Facilitator | Knowledge and Communication | Wanting to be informed or demanding more information | Wanted to get more information and participation Another group of patients described how they did not get information, if they did not ask. They expressed dissatisfaction with the lowlevel of information they received.Some of them did not dare to speak up, while others asked many questions. They expressed the desire to get more information from their physicians. |
| Ekdahl et al. | Healthcare personell factors | Barrier | Knowledge and Communication | No treatment or information involvment of patients | Wanted to get more information and participation Another group of patients described how they did not get information, if they did not ask. They expressed dissatisfaction with the lowlevel of information they received.Some of them did not dare to speak up, while others asked many questions. They expressed the desire to get more information from their physicians. |
| Ekdahl et al. | Patient factors | Barrier | Trust and Power | Submission towards healthcare personnel | Wanted to get more information and participation Another group of patients described how they did not get information, if they did not ask. They expressed dissatisfaction with the lowlevel of information they received.Some of them did not dare to speak up, while others asked many questions. They expressed the desire to get more information from their physicians. |
| Ekdahl et al. | Patient factors | Barrier | Attitude and Behaviour | Passive behaviour | Did not feel a sense of participation—but did not want to, either Some patients expressed that they did not want to participate in their care in any way. They were pleased with a very passive role and entrusted themselves to the hospital care system. |
| Ekdahl et al. | Patient factors | Barrier | Attitude and Behaviour | Not wanting to participate in decision making | Did not feel a sense of participation—but did not want to, either Some patients expressed that they did not want to participate in their care in any way. They were pleased with a very passive role and entrusted themselves to the hospital care system. |
| Ekdahl et al. | Patient factors | Barrier | Attitude and Behaviour | Not wanting to participate in decision making | Int: Would you like to participate more in your care when you get to hospital? Pat: No. Oh no. No, I won’t. They are allowed to do what they want, as long as I get better. I don’t want to participate in anything |
| Ekdahl et al. | Patient factors | Barrier | Trust and Power | Trust towards healthcare personnel | Int: Would you like to participate more in your care when you get to hospital? Pat: No. Oh no. No, I won’t. They are allowed to do what they want, as long as I get better. I don’t want to participate in anything |
| Ekdahl et al. | Patient factors | Barrier | Trust and Power | Trust towards healthcare personnel | Did not feel a sense of participation—but did not want to, either Some patients expressed that they did not want to participate in their care in any way. They were pleased with a very passive role and entrusted themselves to the hospital care system. |
| Ekdahl et al. | Patient factors | Barrier | Trust and Power | Institution of power and/or trust | Did not feel a sense of participation—but did not want to, either Some patients expressed that they did not want to participate in their care in any way. They were pleased with a very passive role and entrusted themselves to the hospital care system. |
| Ekdahl et al. | Patient factors | Barrier | Trust and Power | Institution of power and/or trust | The main theme: the hospital an institution of power When analyzing all the interviews as a whole, a more latent theme emerged that we interpreted as the patients’ perception of the hospital as some kind of an institution of power, both in terms of its competence, its hierarchy and of its doctors as people ranked higher than themselves. |
| Ekdahl et al. | Decision-making interaction factors | Barrier | Knowledge and Communication | Knowledge/Competence asymmetry | The main theme: the hospital an institution of power When analyzing all the interviews as a whole, a more latent theme emerged that we interpreted as the patients’ perception of the hospital as some kind of an institution of power, both in terms of its competence, its hierarchy and of its doctors as people ranked higher than themselves. |
| Ekdahl et al. | Decision-making interaction factors | Barrier | Trust and Power | Asymmetric power relationship | The main theme: the hospital an institution of powerWhen analyzing all the interviews as a whole, a more latent theme emerged that we interpreted as the patients’ perception of the hospital as some kind of an institution of power, both in terms of its competence, its hierarchy and of its doctors as people ranked higher than themselves. |
| Ekdahl et al. | Patient factors | Barrier | Trust and Power | Institution of power and/or trust | the hospital was considered as an institution of power was based on the patients’ perception of the hospital being more competent and, therefore, more able to make wise decisions about care and medical treatment. They felt that ‘‘the hospital’’ made the right decisions and they often expressed great confidence. |
| Ekdahl et al. | Decision-making interaction factors | Barrier | Knowledge and Communication | Knowledge/Competence asymmetry | the hospital was considered as an institution of power was based on the patients’ perception of the hospital being more competent and, therefore, more able to make wise decisions about care and medical treatment. They felt that ‘‘the hospital’’ made the right decisions and they often expressed great confidence. |
| Ekdahl et al. | Patient factors | Barrier | Trust and Power | Institution of power and/or trust | Int: Do you feel you can influence your care, for example, when you are going home? Pat: When I amgoing home? That decision is up to the hospital. The hospital decides. Good luck, they say. Tomorrow you are going to go home in that car. |
| Ekdahl et al. | Healthcare personell factors | Barrier | Knowledge and Communication | No treatment or information involvment of patients | Some patients expressed the view that doctors, especially chief consultants, are important persons at the top of a hierarchical system who do not believe they need to communicate with patients about their care and diagnostic considerations. Especially around discharge, it seemed that the patients were overruled and not always listened to. |
| Ekdahl et al. | Healthcare personell factors | Barrier | Attitude and Behaviour | No decisional involvment of patients | Some patients expressed the view that doctors, especially chief consultants, are important persons at the top of a hierarchical system who do not believe they need to communicate with patients about their care and diagnostic considerations. Especially around discharge, it seemed that the patients were overruled and not always listened to. |
| Ekdahl et al. | Patient factors | Barrier | Trust and Power | Feeling incapacitated | Some patients expressed the view that doctors, especially chief consultants, are important persons at the top of a hierarchical system who do not believe they need to communicate with patients about their care and diagnostic considerations. Especially around discharge, it seemed that the patients were overruled and not always listened to. |
| Ekdahl et al. | Decision-making interaction factors | Barrier | Trust and Power | Asymmetric power relationship | Some patients expressed the view that doctors, especially chief consultants, are important persons at the top of a hierarchical system who do not believe they need to communicate with patients about their care and diagnostic considerations. Especially around discharge, it seemed that the patients were overruled and not always listened to. |
| Ekdahl et al. | Healthcare personell factors | Barrier | Trust and Power | Exercising power and dominance | Some patients expressed the view that doctors, especially chief consultants, are important persons at the top of a hierarchical system who do not believe they need to communicate with patients about their care and diagnostic considerations. Especially around discharge, it seemed that the patients were overruled and not always listened to. |
| Ekdahl et al. | Healthcare personell factors | Barrier | Trust and Power | Being ignored/Ignoring patients | Some patients expressed the view that doctors, especially chief consultants, are important persons at the top of a hierarchical system who do not believe they need to communicate with patients about their care and diagnostic considerations. Especially around discharge, it seemed that the patients were overruled and not always listened to. |
| Ekdahl et al. | Patient factors | Barrier | Trust and Power | Institution of power and/or trust | In spite of these conditions, most of the patients expressed great confidence in the health care they received and did not make too much effort to be informed or try to understand what was happening. |
| Ekdahl et al. | Patient factors | Barrier | Trust and Power | Institution of power and/or trust | Int: When you were in hospital, did you participate in your care and treatment? Pat: My treatment? No, they do what they think they have to do. What they think is the best for me at just that time. |
| Ekdahl et al. | Decision-making interaction factors | Barrier | Knowledge and Communication | Knowledge/Competence asymmetry | Int: When you were in hospital, did you participate in your care and treatment? Pat: My treatment? No, they do what they think they have to do. What they think is the best for me at just that time. |
| Ekdahl et al. | Patient factors | Barrier | Trust and Power | Submission towards healthcare personnel | Int: When you were in hospital, did you participate in your care and treatment? Pat: My treatment? No, they do what they think they have to do. What they think is the best for me at just that time. |
| Ekdahl et al. | Patient factors | Facilitator | Knowledge and Communication | Wanting to express themselves, issue opinions and preferences and to be heard | They want to be listened to when they are explaining how they feel or what they think about their condition, and they want to understand what is happening to them. |
| Ekdahl et al. | Patient factors | Facilitator | Knowledge and Communication | Wanting to be informed or demanding more information | According to the main result of this study participation the patients, first and foremost, means information. Patients want to be informed about investigative procedures, diagnostic considerations, their illnesses, and their treatment, and want better communication concerning their care. |
| Ekdahl et al. | Patient factors | Facilitator | Attitude and Behaviour | Wanting to be involved through decisional participation | According to the main result of this study participation the patients, first and foremost, means information. Patients want to be informed about investigative procedures, diagnostic considerations, their illnesses, and their treatment, and want better communication concerning their care. |
| Ekdahl et al. | Patient factors | Barrier | Attitude and Behaviour | Not wanting to participate in decision making | The patients expressed their wish to be informed thoroughly — but they did not express any wish to take active part in the medical decision making. |
| Ekdahl et al. | Patient factors | Facilitator | Knowledge and Communication | Wanting to express themselves, issue opinions and preferences and to be heard | The majority defined participation as asking questions and/or obtaining information |
| Ekdahl et al. | Patient factors | Barrier | Attitude and Behaviour | Not wanting to participate in decision making | On the basis on our findings, together with the findings of Bastiaens et al. [32], it seems that a majority of elderly patients prefer non-participation in medical decisions, but nevertheless, good information and communication. |
| Ekdahl et al. | Patient factors | Barrier | Health and Age | Being ill | Compromising factors for goodcommunicationare the patient’s own illness, having many persons involved in the patient’s care who do not know the patient and the patient’s preferences, differing treatment strategies amongdoctors, fastpatient turnover in the hospitals, stressed hospital personnel, and linguistic problems with doctors not fluent in the patient’s own language |
| Ekdahl et al. | Healthcare system /organisatonal factors | Barrier | Treatment Organization and Risk | Healthcare staff rotation | Compromising factors for goodcommunicationare the patient’s own illness, having many persons involved in the patient’s care who do not know the patient and the patient’s preferences, differing treatment strategies amongdoctors, fastpatient turnover in the hospitals, stressed hospital personnel, and linguistic problems with doctors not fluent in the patient’s own language |
| Ekdahl et al. | Patient factors | Barrier | Treatment Organization and Risk | Facing diverging treatment strategies | Compromising factors for goodcommunicationare the patient’s own illness, having many persons involved in the patient’s care who do not know the patient and the patient’s preferences, differing treatment strategies amongdoctors, fastpatient turnover in the hospitals, stressed hospital personnel, and linguistic problems with doctors not fluent in the patient’s own language |
| Ekdahl et al. | Healthcare personell factors | Barrier | Treatment Organization and Risk | Facing diverging treatment strategies | Compromising factors for goodcommunicationare the patient’s own illness, having many persons involved in the patient’s care who do not know the patient and the patient’s preferences, differing treatment strategies amongdoctors, fastpatient turnover in the hospitals, stressed hospital personnel, and linguistic problems with doctors not fluent in the patient’s own language |
| Ekdahl et al. | Patient factors | Barrier | Trust and Power | Unknown healthcare provider | Compromising factors for goodcommunicationare the patient’s own illness, having many persons involved in the patient’s care who do not know the patient and the patient’s preferences, differing treatment strategies amongdoctors, fastpatient turnover in the hospitals, stressed hospital personnel, and linguistic problems with doctors not fluent in the patient’s own language |
| Ekdahl et al. | Healthcare system /organisatonal factors | Barrier | Treatment Organization and Risk | High Workload | Compromising factors for goodcommunicationare the patient’s own illness, having many persons involved in the patient’s care who do not know the patient and the patient’s preferences, differing treatment strategies amongdoctors, fastpatient turnover in the hospitals, stressed hospital personnel, and linguistic problems with doctors not fluent in the patient’s own language |
| Ekdahl et al. | Healthcare personell factors | Barrier | Treatment Organization and Risk | Time pressure | Compromising factors for goodcommunicationare the patient’s own illness, having many persons involved in the patient’s care who do not know the patient and the patient’s preferences, differing treatment strategies amongdoctors, fastpatient turnover in the hospitals, stressed hospital personnel, and linguistic problems with doctors not fluent in the patient’s own language |
| Ekdahl et al. | Healthcare personell factors | Barrier | Knowledge and Communication | Linguistic issues | Compromising factors for goodcommunicationare the patient’s own illness, having many persons involved in the patient’s care who do not know the patient and the patient’s preferences, differing treatment strategies amongdoctors, fastpatient turnover in the hospitals, stressed hospital personnel, and linguistic problems with doctors not fluent in the patient’s own language |
| Ekdahl et al. | Patient factors | Barrier | Treatment Organization and Risk | Facing diverging treatment strategies | The differing treatment strategies among doctors and the lack of clear responsibility for treatment decisions, including discharge, make it very difficult for patients to feel they are participating |
| Ekdahl et al. | Healthcare personell factors | Barrier | Treatment Organization and Risk | Facing diverging treatment strategies | The differing treatment strategies among doctors and the lack of clear responsibility for treatment decisions, including discharge, make it very difficult for patients to feel they are participating |
| Ekdahl et al. | Patient factors | Barrier | Knowledge and Communication | Lack of medical or treatment related knowledge | These patients’ physical illnesses and their decline in hearing, vision, and higher cognitive functions are associated with greater effort required to gain information about their health and/or illnesses, for example, by attending meetings or taking advantage of other educational opportunities.Also,due to the cohort effect, it is unlikely that these patients sought information through the Internet; none of our patients expressed spontaneously that they did so. All these factors contribute to poor health literacy in the frail elderly. |
| Ekdahl et al. | Patient factors | Barrier | Trust and Power | Institution of power and/or trust | According to our latent interpretation of the results, we found that the patients perceived the hospital care system as an institution of power with which it is not possible to argue or disagree. The patients focused on the superior competence of health care professionals as the main reason for this power, which they relied on with confidence. |
| Ekdahl et al. | Decision-making interaction factors | Barrier | Knowledge and Communication | Knowledge/Competence asymmetry | According to our latent interpretation of the results, we found that the patients perceived the hospital care system as an institution of power with which it is not possible to argue or disagree. The patients focused on the superior competence of health care professionals as the main reason for this power, which they relied on with confidence. |
| Ekdahl et al. | Patient factors | Barrier | Trust and Power | Trust towards healthcare personnel | According to our latent interpretation of the results, we found that the patients perceived the hospital care system as an institution of power with which it is not possible to argue or disagree. The patients focused on the superior competence of health care professionals as the main reason for this power, which they relied on with confidence. |
| Ekdahl et al. | Decision-making interaction factors | Barrier | Trust and Power | Submission towards healthcare personnel | Int: When you were in hospital, did you participate in your care and treatment? Pat: My treatment? No, they do what they think they have to do. What they think is the best for me at just that time. |
| Ekdahl et al. | Patient factors | Barrier | Trust and Power | Submission towards healthcare personnel | According to our latent interpretation of the results, we found that the patients perceived the hospital care system as an institution of power with which it is not possible to argue or disagree. The patients focused on the superior competence of health care professionals as the main reason for this power, which they relied on with confidence. |
| Ekdahl et al. | Patient factors | Barrier | Health and Age | Being old | Some of our patients clearly expressed that they would have participated more in medical decisions when younger. |
| Ekdahl et al. | Patient factors | Barrier | Health and Age | Being confused | Other patients expressed their own incapacity to participate due to confusion |
| Hamelinck et al. | Patient factors | Facilitator | Attitude and Behaviour | Wanting to be involved through decisional participation | For all decisions, both age groups most frequently preferred a shared role before consultation, except for decisions about aHT, for which younger patients more commonly preferred an active role. |
| Hamelinck et al. | Healthcare personell factors | Barrier | Attitude and Behaviour | No decisional involvment of patients | Older patients more often than younger patients perceived they had not been involved in decisions about systemic therapy. |
| Hamelinck et al. | Patient factors | Facilitator | Attitude and Behaviour | Wanting to be involved through decisional participation | Both younger and older participants most often preferred a shared role (49% and 60%, respectively) before consultation |
| Hamelinck et al. | Patient factors | Barrier | Attitude and Behaviour | Not wanting to participate in decision making | and only 16% of younger and 8% of older participants preferred a passive role. |
| Hamelinck et al. | Patient factors | Facilitator | Attitude and Behaviour | Active behaviour | After consultation, both younger and older participants most frequently reported to have perceived they had had an active role (49% and 56%, respectively), followed by shared (37% and 32%) and passive (14% and 12%) roles. |
| Hamelinck et al. | Healthcare personell factors | Barrier | Attitude and Behaviour | No decisional involvment of patients | The remainder of the younger participants were most often more involved than initially desired (28%), whereas older participants weremost often less involved (27%). |
| Hamelinck et al. | Healthcare personell factors | Barrier | Attitude and Behaviour | No decisional involvment of patients | Also in this decision, younger participants were most often more involved than initially desired (33%) and older participants most often less involved than desired (41%). |
| Hamelinck et al. | Patient factors | Facilitator | Attitude and Behaviour | Wanting to be involved through decisional participation | Only few older patients wished a passive role, and most preferred to make the decision themselves or together with their oncologist, in line with another recent study39 showing that most older patients preferred a shared or active role over a passive role. |
| Hamelinck et al. | Patient factors | Facilitator | Attitude and Behaviour | Wanting to be involved through decisional participation | Our finding that both younger and older patients most often preferred to be involved in making the decision about type of surgery is in line with one of the few other prospective studies among newly diagnosed patients with early-stage disease eligible for BCS and mastectomy |
| Hamelinck et al. | Healthcare personell factors | Barrier | Attitude and Behaviour | No decisional involvment of patients | This is particularly important because clinicians often underestimate patients’ decisional role preferences and rarely ask patients for their preferences |
| Hamelinck et al. | Patient factors | Barrier | Treatment Organization and Risk | Treatment related dismissal of decisional involvment | Although decisional role preferences did not significantly differ between age groups, preferred roles in deciding whether to undergo aHT stand out, with relatively more younger than older patients preferring to make the decision themselves. |
| Hamelinck et al. | Decision-making interaction factors | Barrier | Treatment Organization and Risk | Treatment related dismissal of decisional involvment | In contrast, older patients more often than younger patients felt that they had not been involved in making the decision concerning aCT. The treatment guidelines indeed state that aCT may not be a reasonable treatment option for patients over 70 years of age.34 Similarly, older patients more often perceived to have had a passive role in deciding about aHT. In clinical practice, patients with HR-positive tumors, irrespective of their age, are rarely offered a choice about aHT. |
| Hamelinck et al. | Healthcare personell factors | Barrier | Treatment Organization and Risk | Treatment related dismissal of decisional involvment | In contrast, older patients more often than youngerpatients felt that they had not been involved in making the decision concerning aCT. The treatment guidelines indeed state that aCT may not be a reasonable treatment option for patients over 70 years of age.34 Similarly, older patients more often perceived to have had a passive role in deciding about aHT. In clinical practice, patients with HR-positive tumors, irrespective of their age, are rarely offered a choice about aHT. |
| Hamelinck et al. | Patient factors | Facilitator | Attitude and Behaviour | Wanting to be involved through decisional participation | It is important for clinicians to know that most older patients are willing to be involved in decision making. However, we also want to stress the variation in role preferences among older patients and across the different decisions. |
| Hamelinck et al. | Patient factors | Barrier | Treatment Organization and Risk | Treatment related dismissal of decisional involvment | It is important for clinicians to know that most older patients are willing to be involved in decision making. However, we also want to stress the variation in role preferences among older patients and across the different decisions. |
| Mandelblatt et al. | Patient factors | Facilitator | Treatment Organization and Risk | Treatment satisfaction | Greater SDM was also associated with improved short-term satisfaction. |
| Mandelblatt et al. | Patient factors | Facilitator | Knowledge and Communication | Supporting family involvment | Of note, women who reported that someone accompanied them to treatment appointments also reported significantly higher SDM than women who went to appointments by themselves |
| Mandelblatt et al. | Patient factors | Facilitator | Attitude and Behaviour | Active behaviour | Women who sought information from someone other than their physicians also reported greater SDM than women who relied on physicians alone for information |
| Mandelblatt et al. | Patient factors | Facilitator | Knowledge and Communication | Being offered a choice | Finally, women who reported having a treatment choice reported significantly higher SDM levels than those who felt they did not have a choice. |
| Mandelblatt et al. | Patient factors | Barrier | Health and Age | Being old | Interestingly, there was a significant interaction between age and SDM, with SDM only being significantly related to adjuvant treatment among women ages 67 to 74, and not among women 75 years and older |
| Mandelblatt et al. | Patient factors | Facilitator | Treatment Organization and Risk | Treatment satisfaction | Women who rated SDM about their treatment more highly reported greater short-term global satisfaction with their treatment, after considering age, treatment received, and education |
| Mandelblatt et al. | Patient factors | Facilitator | Knowledge and Communication | Supporting family involvment | We found that older women who brought others into their treatment consultation and those who were information seekers were likely to report high levels of SDM |
| Hamelinck et al. | Patient factors | Facilitator | Attitude and Behaviour | Wanting to be involved through decisional participation | We found that older women who brought others into their treatment consultation and those who were information seekers were likely to report high levels of SDM |
| Hamelinck et al. | Patient factors | Barrier | Treatment Organization and Risk | Treatment related dismissal of decisional involvment | Interestingly, SDM was not associated with the choice between MST and BCS, but was associated with the more controversial use of adjuvant treatment |
| Mandelblatt et al. | Patient factors | Facilitator | Treatment Organization and Risk | Treatment satisfaction | Use of SDM appears to be associated with patient satisfaction with treatment. However, SDM had a negative association with impact on women’s lives. |
| Mandelblatt et al. | Patient factors | Facilitator | Treatment Organization and Risk | Treatment satisfaction | Our results show that more SDM is associated with greater satisfaction with treatment. |
| Gainer et al. | Patient factors | Facilitator | Knowledge and Communication | Wanting to be informed or demanding more information | Patient groups expressed a desire to receive information earlier in their care to allow time to identify personal values and preferences in developing plans for treatment. |
| Gainer et al. | Patient factors | Facilitator | Attitude and Behaviour | Wanting to be involved through decisional participation | Patient groups expressed a desire to receive information earlier in their care to allow time to identify personal values and preferences in developing plans for treatment. |
| Gainer et al. | Patient factors | Facilitator | Knowledge and Communication | Wanting to express themselves, issue opinions and preferences and to be heard | Patient groups expressed a desire to receive information earlier in their care to allow time to identify personal values and preferences in developing plans for treatment. |
| Gainer et al. | Decision-making interaction factors | Facilitator | Treatment Organization and Risk | Formal SDM approach | Both groups strongly supported a formal approach for shared decision making with a decisional coach to provide information and facilitate communication with the care team. |
| Gainer et al. | Healthcare system /organisatonal factors | Facilitator | Treatment Organization and Risk | Formal SDM approach | Both groups strongly supported a formal approach for shared decision making with a decisional coach to provide information and facilitate communication with the care team. |
| Gainer et al. | Decision-making interaction factors | Facilitator | Treatment Organization and Risk | SDM mediator | Both groups strongly supported a formal approach forshared decision making with a decisional coach to provide information and facilitate communication with the care team. |
| Gainer et al. | Healthcare system /organisatonal factors | Facilitator | Health and Age | Need for individualized care - Treatment complexity and multimorbid patients | Given the trend toward older and frail patients referred for complex cardiac procedures, the need for an effective shared decision making process is compelling |
| Gainer et al. | Healthcare personell factors | Barrier | Knowledge and Communication | Linguistic issues | Patient groups identified structural and conceptual barriers with the consent form document used before treatment. Structural issues included consent form language complexity and small font |
| Gainer et al. | Patient factors | Barrier | Health and Age | Forgetting discussions or given information | Very few of the patients recollected any aspect of the standard form when shown |
| Gainer et al. | Patient factors | Barrier | Health and Age | Forgetting discussions or given information | I’m wondering if you remember this form, this is the consent form to have cardiac surgery. Would you have read through it, is it something – Patient 4: No, I’ve never seen it before. [P3, P1, P2 ‘‘That’s true’’,‘‘I’ve never seen it’’] |
| Gainer et al. | Patient factors | Barrier | Attitude and Behaviour | Not wanting to participate in decision making | Surgeon 3: Having consented many patients for high-risk studies in sepsis, where we tell them up front the mortality is 40%, they just say where do I sign? We’ve got an 18-page consent form and they go straight to the bottom line |
| Gainer et al. | Patient factors | Barrier | Attitude and Behaviour | Passive behaviour | Surgeon 3: Having consented many patients for high-risk studies in sepsis, where we tell them up front the mortality is 40%, they just say where do I sign? We’ve got an 18-page consent form and they go straight to the bottom line |
| Gainer et al. | Healthcare personell factors | Facilitator | Treatment Organization and Risk | Alternative choices / Ambiguity | Surgeon 3: The document, I don’t think, has anything to do with decision making. By the time you get to that the document the decision has been made. I don’t often find myself helping patients make decisions. Unless there is ambiguity about what the right way to go is |
| Gainer et al. | Patient factors | Barrier | Trust and Power | Feeling powerless / Having no control | Patient 3: I think, to a point, this is out of my hands. What will be, will be. I’m just along for the ride. |
| Gainer et al. | Patient factors | Barrier | Trust and Power | Feeling powerless / Having no control | Patient 5: Yeah, I think knowing these numbers would help me be better prepared. It may not impact the surgery but, down the road, I think I’d feel like I’d have a bit more awareness; control. |
| Gainer et al. | Patient factors | Barrier | Trust and Power | Feeling powerless / Having no control | I think younger people may question..you know, if you are 40 or 50 or 60; but once you start getting over 75, it’s like they just say.the doctor says I need it.so they will just do it. |
| Gainer et al. | Patient factors | Barrier | Health and Age | Being old | I think younger people may question..you know, if you are 40 or 50 or 60; but once you start getting over 75, it’s like they just say.the doctor says I need it.so they will just do it. |
| Gainer et al. | Healthcare personell factors | Barrier | Trust and Power | Exercising power and dominance | I think younger people may question..you know, if you are 40 or 50 or 60; but once you start getting over 75, it’s like they just say.the doctor says I need it.so they will just do it. |
| Gainer et al. | Patient factors | Barrier | Trust and Power | Submissive behaviour | Surgeon 4: I can’t remember the last time that an over 70-y-old really asked a relevant question. Unless they are just trying to be polite, but really they’re just looking for somebody to tell them what the best thing to do is. |
| Gainer et al. | Patient factors | Barrier | Trust and Power | Submission towards healthcare personnel | Surgeon 4: I can’t remember the last time that an over 70-y-old really asked a relevant question. Unless they are just trying to be polite, but really they’re just looking for somebody to tell them what the best thing to do is. |
| Gainer et al. | Patient factors | Barrier | Attitude and Behaviour | Not wanting to participate in decision making | Surgeon 4: I can’t remember the last time that an over 70-y-old really asked a relevant question. Unless they are just trying to be polite, but really they’re just looking for somebody to tell them what the best thing to do is. |
| Gainer et al. | Patient factors | Barrier | Attitude and Behaviour | Depending on family or healthcare personnel | Surgeon 4: I can’t remember the last time that an over 70-y-old really asked a relevant question. Unless they are just trying to be polite, but really they’re just looking for somebody to tell them what the best thing to do is. |
| Gainer et al. | Decision-making interaction factors | Barrier | Trust and Power | Submission towards healthcare personnel | Patient 5: Once I’m there, I’m in their hands. It’s up to them how things are going to go. |
| Gainer et al. | Patient factors | Barrier | Trust and Power | Submission towards healthcare personnel | Patient 5: Once I’m there, I’m in their hands. It’s up to them how things are going to go. |
| Gainer et al. | Patient factors | Barrier | Trust and Power | Submissive behaviour | Patient 5: Once I’m there, I’m in their hands. It’s up to them how things are going to go. |
| Gainer et al. | Patient factors | Barrier | Trust and Power | Feeling powerless / Having no control | Patient 5: Once I’m there, I’m in their hands. It’s up to them how things are going to go. |
| Gainer et al. | Patient factors | Barrier | Attitude and Behaviour | Depending on family or healthcare personnel | Patient 5: Once I’m there, I’m in their hands. It’s up to them how things are going to go. |
| Gainer et al. | Patient factors | Barrier | Attitude and Behaviour | Depending on family or healthcare personnel | Patients noted external and internal locus of control issues when discussing demand for information regarding surgery and engagement in the consent process. Nurse and physician groups expressed that older patients typically showed a tendency to externalize the locus of control through deference to physicians, less direct engagement in asking questions to the care team, and heavier reliance on family advocates: |
| Gainer et al. | Patient factors | Barrier | Trust and Power | Feeling powerless / Having no control | Patients noted external and internal locus of control issues when discussing demand for information regarding surgery and engagement in the consent process. Nurse and physician groups expressed that older patients typically showed a tendency to externalize the locus of control through deference to physicians, less direct engagement in asking questions to the care team, and heavier reliance on family advocates: |
| Gainer et al. | Patient factors | Barrier | Attitude and Behaviour | Passive behaviour | Patients noted external and internal locus of control issues when discussing demand for information regarding surgery and engagement in the consent process. Nurse and physician groups expressed that older patients typically showed a tendency to externalize the locus of control through deference to physicians, less direct engagement in asking questions to the care team, and heavier reliance on family advocates: |
| Gainer et al. | Patient factors | Barrier | Trust and Power | Submissive behaviour | Patients noted external and internal locus of control issues when discussing demand for information regarding surgery and engagement in the consent process. Nurse and physician groups expressed that older patients typically showed a tendency to externalize the locus of control through deference to physicians, less direct engagement in asking questions to the care team, and heavier reliance on family advocates: |
| Gainer et al. | Healthcare personell factors | Barrier | Treatment Organization and Risk | Time pressure | Time was expressed as a significant barrier in providing cohesive, relevant and meaningful education in a way that would provide patients with the tools necessary to fully comprehend the nature of their disease, the risk, benefits, and options |
| Gainer et al. | Healthcare system /organisatonal factors | Barrier | Treatment Organization and Risk | Scheduling issue | Time was expressed as a significant barrier in providing cohesive, relevant and meaningful education in a way that would provide patients with the tools necessary to fully comprehend the nature of their disease, the risk, benefits, and options |
| Gainer et al. | Patient factors | Barrier | Knowledge and Communication | Lack of medical or treatment related knowledge | Time was expressed as a significant barrier in providing cohesive, relevant and meaningful education in a way that would provide patients with the tools necessary to fully comprehend the nature of their disease, the risk, benefits, and options |
| Gainer et al. | Healthcare system /organisatonal factors | Barrier | Treatment Organization and Risk | Scheduling issue | Surgeon 2: We’re thrust into that situation all the time. The schedule gets changed, we go to 6.2 to find a morbidly obese diabetic with hypertrophic ventricle and poor distal vessels. And the night before surgery, we start the conversation. That’s the type of thing you’d like to have the family in the office 2 months beforehand. |
| Gainer et al. | Patient factors | Barrier | Knowledge and Communication | Lack of medical or treatment related knowledge | Focus group participants expressed that patients are hesitant to communicate when they do not understand information. |
| Gainer et al. | Decision-making interaction factors | Barrier | Knowledge and Communication | Linguistic issues | Focus group participants expressed that patients are hesitant to communicate when they do not understand information. |
| Gainer et al. | Healthcare personell factors | Barrier | Knowledge and Communication | Linguistic issues | Focus group participants expressed that patients are hesitant to communicate when they do not understand information. |
| Gainer et al. | Healthcare system /organisatonal factors | Barrier | Treatment Organization and Risk | Scheduling issue | Time was seen as crucial for any other part of the education to be effective. This includes both the length of time allocated to the discussion and the point in time at which the discussion takes place. Patients were careful to note that, although decision aids were a welcome addition, if they were not deployed earlier in the care process, it would result in little change to patient education and engagement. |
| Gainer et al. | Healthcare personell factors | Barrier | Treatment Organization and Risk | Time pressure | Time was seen as crucial for any other part of the education to be effective. This includes both the length of time allocated to the discussion and the point in time at which the discussion takes place. Patients were careful to note that, although decision aids were a welcome addition, if they were not deployed earlier in the care process, it would result in little change to patient education and engagement. |
| Gainer et al. | Healthcare system /organisatonal factors | Barrier | Treatment Organization and Risk | Scheduling issue | Patient 1: I think even with this here the day before surgery, you know, even with this form here and the day before or same day as the surgery, it’s not going to give you time to consider it. |
| Gainer et al. | Healthcare system /organisatonal factors | Barrier | Treatment Organization and Risk | Scheduling issue | Patient 2: I think I would be more inclined to read it if I had the time to read it. |
| Ekdahl et al. | Healthcare personell factors | Barrier | Trust and Power | Being ignored/Ignoring patients | Patient 5: Let me get this straight, they were fantastic up there, the nurses and the doctors, and they just work right off their feet. But it just felt that, I was left out, they were talking about me. |
| Gainer et al. | Healthcare personell factors | Barrier | Attitude and Behaviour | No decisional involvment of patients | Patient 5: Let me get this straight, they were fantastic up there, the nurses and the doctors, and they just work right off their feet. But it just felt that, I was left out, they were talking about me. |
| Gainer et al. | Healthcare personell factors | Barrier | Knowledge and Communication | No treatment or information involvment of patients | Patient 5: Let me get this straight, they were fantastic up there, the nurses and the doctors, and they just work right off their feet. But it just felt that, I was left out, they were talking about me. |
| Gainer et al. | Patient factors | Facilitator | Attitude and Behaviour | Wanting to be involved through decisional participation | Patients desired personal autonomy through a greater opportunity for individual choice; however, the perception of an asymmetric power relationship with physicians seemed to overshadow these expressions of personal autonomy. |
| Gainer et al. | Patient factors | Facilitator | Knowledge and Communication | Wanting to be informed or demanding more information | Patients desired personal autonomy through a greater opportunity for individual choice; however, the perception of an asymmetric power relationship with physicians seemed to overshadow these expressions of personal autonomy. |
| Gainer et al. | Decision-making interaction factors | Barrier | Trust and Power | Asymmetric power relationship | Patients desired personal autonomy through a greater opportunity for individual choice; however, the perception of an asymmetric power relationship with physicians seemed to overshadow these expressions of personal autonomy. |
| Gainer et al. | Decision-making interaction factors | Barrier | Trust and Power | Asymmetric power relationship | Patient 1: You’re just part of the assembly line, you might slow the assembly line down, stall it a little bit or something. |
| Gainer et al. | Healthcare personell factors | Barrier | Trust and Power | Exercising power and dominance | Patient 1: You’re just part of the assembly line, you might slow the assembly line down, stall it a little bit or something. |
| Gainer et al. | Patient factors | Barrier | Trust and Power | Submissive behaviour | Patient 1: You’re just part of the assembly line, you might slow the assembly line down, stall it a little bit or something. |
| Gainer et al. | Patient factors | Barrier | Trust and Power | Submissive communication semantics | Patient 1: You’re just part of the assembly line, you might slow the assembly line down, stall it a little bit or something. |
| Gainer et al. | Patient factors | Barrier | Attitude and Behaviour | Depending on family or healthcare personnel | Decision making was centered on developing trust and faith in the surgeon rather than weighing different decision options. Nurse and physician groups indicated the passivity of patients could be partly attributed to their perception of little to no choice in their options. |
| Gainer et al. | Patient factors | Barrier | Attitude and Behaviour | Passive behaviour | Decision making was centered on developing trust and faith in the surgeon rather than weighing different decision options. Nurse and physician groups indicated the passivity of patients could be partly attributed to their perception of little to no choice in their options. |
| Gainer et al. | Patient factors | Barrier | Attitude and Behaviour | Not having a choice | Decision making was centered on developing trust and faith in the surgeon rather than weighing different decision options. Nurse and physician groups indicated the passivity of patients could be partly attributed to their perception of little to no choice in their options. |
| Gainer et al. | Decision-making interaction factors | Barrier | Knowledge and Communication | Knowledge/Competence asymmetry | Decision making was centered on developing trust and faith in the surgeon rather than weighing different decision options. Nurse and physician groups indicated the passivity of patients could be partly attributed to their perception of little to no choice in their options. |
| Gainer et al. | Patient factors | Barrier | Attitude and Behaviour | Not having a choice | Nurse 4: Not uncommon for them to say, ‘‘I don’t have any choice.’’ You say, ‘‘So are you ready to go ahead?’’ and they say, ‘‘Well, I don’t have a choice.’’ I think a lot of them probably don’t even realize that they can refuse. They just come in and say, this is what the doctor says, I am going to sign it, the doctor says. |
| Gainer et al. | Patient factors | Barrier | Trust and Power | Submission towards healthcare personnel | Nurse 4: Not uncommon for them to say, ‘‘I don’t have any choice.’’ You say, ‘‘So are you ready to go ahead?’’ and they say, ‘‘Well, I don’t have a choice.’’ I think a lot of them probably don’t even realize that they can refuse. They just come in and say, this is what the doctor says, I am going to sign it, the doctor says. |
| Gainer et al. | Patient factors | Barrier | Attitude and Behaviour | Passive behaviour | Nurse 4: Not uncommon for them to say, ‘‘I don’t have any choice.’’ You say, ‘‘So are you ready to go ahead?’’ and they say, ‘‘Well, I don’t have a choice.’’ I think a lot of them probably don’t even realize that they can refuse. They just come in and say, this is what the doctor says, I am going to sign it, the doctor says. |
| Gainer et al. | Patient factors | Barrier | Attitude and Behaviour | Not having a choice | Surgeon 5: I think patients in hospital feel pressured that there is only one option. They don’t feel there is a choice. |
| Gainer et al. | Patient factors | Barrier | Trust and Power | Submissive behaviour | Surgeon 5: I think patients in hospital feel pressured that there is only one option. They don’t feel there is a choice. |
| Gainer et al. | Patient factors | Barrier | Knowledge and Communication | Prior missinformation through family, friends, internet or other sources | Expectations of surgery and its outcomes came from a number of sources, such as friends or family members experiencing similar cardiac events, media coverage, or reviews online |
| Gainer et al. | Patient factors | Barrier | Knowledge and Communication | Prior missinformation through family, friends, internet or other sources | Nurse 1: Even an uncomplicated patient doing well. they had no idea they would have a breathing tube in and that they would be waking up, etc, just the routine course of an uncomplicated recovery. they don’t know anything about what comes after. |
| Gainer et al. | Patient factors | Barrier | Knowledge and Communication | Lack of medical or treatment related knowledge | Nurse 1: Even an uncomplicated patient doing well. they had no idea they would have a breathing tube in and that they would be waking up, etc, just the routine course of an uncomplicated recovery. they don’t know anything about what comes after. |
| Gainer et al. | Patient factors | Barrier | Knowledge and Communication | Lack of medical or treatment related knowledge | Nurse 3: Especially given what they say afterwards if they are a month in to recovery and they wished they had never done it, had they known. ‘‘if I had known what this was going to be like. I would have never gone through it.’’ |
| Gainer et al. | Patient factors | Barrier | Knowledge and Communication | No treatment or information involvment of patients | Nurse 3: Especially given what they say afterwards if they are a month in to recovery and they wished they had never done it, had they known. ‘‘if I had known what this was going to be like. I would have never gone through it.’’ |
| Gainer et al. | Patient factors | Barrier | Knowledge and Communication | Lack of medical or treatment related knowledge | Nurse 6: We just had a surgical case that resulted in a stroke postoperatively. The family was upset because, even though they had the consent, they said ‘our father did sign up for surgery, but he didn’t sign up for a stroke’. |
| Gainer et al. | Patient factors | Barrier | Knowledge and Communication | No treatment or information involvment of patients | Nurse 6: We just had a surgical case that resulted in a stroke postoperatively. The family was upset because, even though they had the consent, they said ‘our father did sign up for surgery, but he didn’t sign up for a stroke’. |
| Gainer et al. | Patient factors | Barrier | Knowledge and Communication | Lack of medical or treatment related knowledge | Nurses stated that better communication and education preoperatively would have to occur in order for patients and their families to reorient unrealistic expectations towards something more in line with scenarios they could experience following cardiac surgery: |
| Gainer et al. | Patient factors | Barrier | Knowledge and Communication | No treatment or information involvment of patients | Nurses stated that better communication and education preoperatively would have to occur in order for patients and their families to reorient unrealistic expectations towards something more in line with scenarios they could experience following cardiac surgery: |
| Gainer et al. | Healthcare personell factors | Barrier | Treatment Organization and Risk | Time pressure | Without sufficient time to educate and realign expectations of care, patients tended to generate erroneous expectations for their treatment based on anecdotal evidence, such as personal family experience |
| Gainer et al. | Patient factors | Barrier | Knowledge and Communication | Lack of medical or treatment related knowledge | Without sufficient time to educate and realign expectations of care, patients tended to generate erroneous expectations for their treatment based on anecdotal evidence, such as personal family experience |
| Gainer et al. | Patient factors | Barrier | Knowledge and Communication | Prior missinformation through family, friends, internet or other sources | Without sufficient time to educate and realign expectations of care, patients tended to generate erroneous expectations for their treatment based on anecdotal evidence, such as personal family experience |
| Gainer et al. | Patient factors | Barrier | Knowledge and Communication | Prior missinformation through family, friends, internet or other sources | Patient 4: With my sister, they put a valve in that was too small, I thought ‘well, the same thing will happen to me.’ |
| Gainer et al. | Patient factors | Barrier | Knowledge and Communication | Prior missinformation through family, friends, internet or other sources | Patient 2: I knew what to expect because my mother had it done 4 y ago. |
| Gainer et al. | Decision-making interaction factors | Barrier | Trust and Power | Asymmetric power relationship | Patients expressed the importance of having family members or close friends to attend the consent process. Patients would depend on advocates to acquire information and ask questions but also to act as a third party between the patient and the physician. |
| Gainer et al. | Patient factors | Barrier | Attitude and Behaviour | Depending on family or healthcare personnel | Patients expressed the importance of having family members or close friends to attend the consent process. Patients would depend on advocates to acquire information and ask questions but also to act as a third party between the patient and the physician. |
| Gainer et al. | Patient factors | Barrier | Trust and Power | Feeling powerless / Having no control | Patients expressed the importance of having family members or close friends to attend the consent process. Patients would depend on advocates to acquire information and ask questions but also to act as a third party between the patient and the physician. |
| Gainer et al. | Decision-making interaction factors | Barrier | Trust and Power | Asymmetric power relationship | Patient 4: My brother got information any time he wanted. I mean, he’s a well-educated guy and he knew what questions to ask, unlike me. So, I mean, he was there, he was in my corner, that always helped, knowing that. |
| Gainer et al. | Patient factors | Barrier | Attitude and Behaviour | Depending on family or healthcare personnel | Patient 4: My brother got information any time he wanted. I mean, he’s a well-educated guy and he knew what questions to ask, unlike me. So, I mean, he was there, he was in my corner, that always helped, knowing that. |
| Gainer et al. | Patient factors | Barrier | Trust and Power | Feeling powerless / Having no control | Patient 4: My brother got information any time he wanted. I mean, he’s a well-educated guy and he knew what questions to ask, unlike me. So, I mean, he was there, he was in my corner, that always helped, knowing that. |
| Gainer et al. | Patient factors | Barrier | Knowledge and Communication | Lack of medical or treatment related knowledge | Patient 4: My brother got information any time he wanted. I mean, he’s a well-educated guy and he knew what questions to ask, unlike me. So, I mean, he was there, he was in my corner, that always helped, knowing that. |
| Gainer et al. | Patient factors | Barrier | Trust and Power | Submissive communication semantics | Patient 4: My brother got information any time he wanted. I mean, he’s a well-educated guy and he knew what questions to ask, unlike me. So, I mean, he was there, he was in my corner, that always helped, knowing that. |
| Gainer et al. | Patient factors | Barrier | Attitude and Behaviour | Depending on family or healthcare personnel | Surgeon 3: I get shivers when I only meet the patient and there is not a family member. Where are they? I get very uncomfortable and all my bad experiences have been alone. |
| Gainer et al. | Decision-making interaction factors | Facilitator | Treatment Organization and Risk | SDM mediator | Nurse 1: At another hospital I worked at, they had a coordinator who would organize the operations and talk to every patient beforehand. Give them a tour where the ICU is, what it looks like, etc. I think the visit made a huge difference. Wherever you could fit that in would be a good start. You could hit on a few points, ‘remember we discussed this, you have the tube in a little longer’. Patients remembered that. |
| Gainer et al. | Decision-making interaction factors | Facilitator | Treatment Organization and Risk | SDM mediator | All groups were also interested in adding a decisional coach into the process of care. The decisional coach would be someone (e.g., a nurse) who could prepare patients and families preoperatively to provide continuity, understand patient-specific values and goals of care, and relay issues to physicians in preparation for the patient–physician encounter. |
| Gainer et al. | Healthcare system /organisatonal factors | Barrier | Treatment Organization and Risk | Healthcare staff rotation | All groups were also interested in adding a decisional coach into the process of care. The decisional coach would be someone (e.g., a nurse) who could prepare patients and families preoperatively to provide continuity, understand patient-specific values and goals of care, and relay issues to physicians in preparation for the patient–physician encounter. |
| Gainer et al. | Decision-making interaction factors | Facilitator | Treatment Organization and Risk | SDM mediator | Core findings found that both patients and providers would prefer consent and access to decisional supports earlier, and a decisional coach who would meet with patients and families to act as a navigator through the course of care (both groups identified an RN as the most appropriate choice to serve as decisional coach). |
| Gainer et al. | Healthcare system /organisatonal factors | Barrier | Treatment Organization and Risk | Scheduling issue | Core findings found that both patients and providers would prefer consent and access to decisional supports earlier, and a decisional coach who would meet with patients and families to act as a navigator through the course of care (both groups identified an RN as the most appropriate choice to serve as decisional coach). |
| Gainer et al. | Healthcare system /organisatonal factors | Barrier | Treatment Organization and Risk | Scheduling issue | Both patient and provider groups identified a lack of adequate time as a major barrier to an optimal informed consent process. Provider groups indicated that meaningful engagement in decision making by patients and families is best achieved by involving patients and families both earlier in the patient’s course and over multiple conversations as compared with a single, typically brief, encounter. |
| Gainer et al. | Healthcare system /organisatonal factors | Barrier | Treatment Organization and Risk | Scheduling issue | Thus, our data strongly support earlier engagement of patients and families over multiple conversations to allow meaningful engagement in decision making. |
| Gainer et al. | Decision-making interaction factors | Facilitator | Treatment Organization and Risk | SDM mediator | All groups in our study were strongly supportive of the addition of an RN decisional coach to guide patients and families before surgery |
| Gainer et al. | Decision-making interaction factors | Facilitator | Treatment Organization and Risk | SDM mediator | Both patients and providers expressed that an RN would be best suited to fulfilling the role of a decisional coach, and serve as an effective advocate in engaging patients |
| Gainer et al. | Patient factors | Facilitator | Attitude and Behaviour | Confidence in participating in decisional involvment | For those in groups who felt they had control over their health, patients were typically more supportive of receiving additional materials preoperatively to supplement the decision making process than patients who externalized control and deferred decision making to their care team. Patients with an internal locus of health control are typically better informed about their disease process |
| Gainer et al. | Patient factors | Barrier | Trust and Power | Feeling powerless / Having no control | For those in groups who felt they had control over their health, patients were typically more supportive of receiving additional materials preoperatively to supplement the decision making process than patients who externalized control and deferred decision making to their care team. Patients with an internal locus of health control are typically better informed about their disease process |
| Gainer et al. | Patient factors | Facilitator | Knowledge and Communication | Adequate medical knowledge | For those in groups who felt they had control over their health, patients were typically more supportive of receiving additional materials preoperatively to supplement the decision making process than patients who externalized control and deferred decision making to their care team. Patients with an internal locus of health control are typically better informed about their disease process |
| Gainer et al. | Patient factors | Barrier | Knowledge and Communication | Lack of medical or treatment related knowledge | For those in groups who felt they had control over their health, patients were typically more supportive of receiving additional materials preoperatively to supplement the decision making process than patients who externalized control and deferred decision making to their care team. Patients with an internal locus of health control are typically better informed about their disease process |
| Huetteman et al. | Healthcare personell factors | Facilitator | Treatment Organization and Risk | Alternative choices / Ambiguity | Understanding patient preferences for shared decision-making is valuable for surgeons to advance patient-centered care, particularly in cases where there is not a clearly superior treatment option, like distal radius fracture (DRF). |
| Huetteman et al. | Patient factors | Barrier | Trust and Power | Trust towards healthcare personnel | Most patients placed distinct trust in the recommendations of hand specialists regarding the technical aspects of the treatment. |
| Huetteman et al. | Patient factors | Facilitator | Knowledge and Communication | Wanting to express themselves, issue opinions and preferences and to be heard | Nonetheless, respondents wanted to provide input when decisions pertained to outcomes or functionality. |
| Huetteman et al. | Patient factors | Facilitator | Attitude and Behaviour | Wanting to be involved through decisional participation | Despite conflicting evidence, most older adult patients desire a shared approach when making treatment decisions |
| Huetteman et al. | Healthcare personell factors | Barrier | Trust and Power | Exercising power and dominance | Of the participants who were not randomized to receive a particular treatment as a part of WRIST (N=15), seven (46%) described that the surgeon alone settled on the final treatment option. These subjects explained that they were either told that they needed surgery (N=5) or only required a cast or splint (N=2). |
| Huetteman et al. | Patient factors | Facilitator | Attitude and Behaviour | Wanting to be involved through decisional participation | One respondent, a 74-year-old female who received a cast, was displeased, noting she would have elected a surgical treatment route if given the choice. |
| Huetteman et al. | Patient factors | Barrier | Attitude and Behaviour | Not having a choice | One respondent, a 74-year-old female who received a cast, was displeased, noting she would have elected a surgical treatment route if given the choice. |
| Huetteman et al. | Patient factors | Barrier | Attitude and Behaviour | Depending on family or healthcare personnel | Others were accepting of the physician’s paternalistic approach (Table 3a). They detailed the ease associated with not needing to consider the options critically. |
| Huetteman et al. | Patient factors | Barrier | Knowledge and Communication | Ease of non-involvment | Others were accepting of the physician’s paternalistic approach (Table 3a). They detailed the ease associated with not needing to consider the options critically. |
| Huetteman et al. | Patient factors | Barrier | Treatment Organization and Risk | Treatment related dismissal of decisional involvment | Subjects who previously underwent treatment for more serious conditions, breast cancer, for instance, clarified that their decision preferences for a DRF were dissimilar to those for that more serious condition (Table 3b). Despite having multiple options for DRF treatment, a fracture was perceived as more straightforward than other conditions; thus, less of an active role in decision-making was desired. |
| Huetteman et al. | Patient factors | Barrier | Attitude and Behaviour | Not wanting to participate in decision making | Subjects who previously underwent treatment for more serious conditions, breast cancer, for instance, clarified that their decision preferences for a DRF were dissimilar to those for that more serious condition (Table 3b). Despite having multiple options for DRF treatment, a fracture was perceived as more straightforward than other conditions; thus, less of an active role in decision-making was desired. |
| Huetteman et al. | Patient factors | Barrier | Knowledge and Communication | Ease of non-involvment | Conversely, one respondent reported she felt frustrated that the surgeon did not suggest a superior option (Table 4b). The subject, a 65-year-old female who elected VLPS, elaborated that she had difficulty deciding on her own because she was not able to think clearly given the amount of pain she was in and relied on her daughter to decide. |
| Huetteman et al. | Patient factors | Barrier | Health and Age | Being ill | Conversely, one respondent reported she felt frustrated that the surgeon did not suggest a superior option (Table 4b). The subject, a 65-year-old female who elected VLPS, elaborated that she had difficulty deciding on her own because she was not able to think clearly given the amount of pain she was in and relied on her daughter to decide. |
| Huetteman et al. | Patient factors | Barrier | Attitude and Behaviour | Depending on family or healthcare personnel | Conversely, one respondent reported she felt frustrated that the surgeon did not suggest a superior option (Table 4b). The subject, a 65-year-old female who elected VLPS, elaborated that she had difficulty deciding on her own because she was not able to think clearly given the amount of pain she was in and relied on her daughter to decide. |
| Huetteman et al. | Patient factors | Barrier | Health and Age | Being in pain | Conversely, one respondent reported she felt frustrated that the surgeon did not suggest a superior option (Table 4b). The subject, a 65-year-old female who elected VLPS, elaborated that she had difficulty deciding on her own because she was not able to think clearly given the amount of pain she was in and relied on her daughter to decide. |
| Huetteman et al. | Patient factors | Barrier | Attitude and Behaviour | Not wanting to participate in decision making | Conversely, one respondent reported she felt frustrated that the surgeon did not suggest a superior option (Table 4b). The subject, a 65-year-old female who elected VLPS, elaborated that she had difficulty deciding on her own because she was not able to think clearly given the amount of pain she was in and relied on her daughter to decide. |
| Huetteman et al. | Patient factors | Barrier | Attitude and Behaviour | Not wanting to participate in decision making | Two of the WRIST participants described a similar desire for surgeon-led decisions. Both suggested that part of the reason they partook in the surgical trial was because they did not want to make the decision alone (Table 5). These subjects indicated that having the computer pick an option for them at random was more desirable than picking themselves. |
| Huetteman et al. | Patient factors | Barrier | Knowledge and Communication | Ease of non-involvment | Two of the WRIST participants described a similar desire for surgeon-led decisions. Both suggested that part of the reason they partook in the surgical trial was because they did not want to make the decision alone (Table 5). These subjects indicated that having the computer pick an option for them at random was more desirable than picking themselves. |
| Huetteman et al. | Patient factors | Facilitator | Attitude and Behaviour | Wanting to be involved through decisional participation | Furthermore, when subjects in the WRIST group were asked about the role they would have wanted if they had had a choice, almost all (N=13) described a mutualistic approach (Table 6b). |
| Huetteman et al. | Patient factors | Facilitator | Attitude and Behaviour | Wanting to be involved through decisional participation | Some of our oldest participants were the most adamant about dictating their care. |
| Huetteman et al. | Patient factors | Facilitator | Trust and Power | Exercising power and dominance | Some of our oldest participants were the most adamant about dictating their care. |
| Huetteman et al. | Patient factors | Facilitator | Knowledge and Communication | Wanting to be informed or demanding more information | Some respondents did note that answers to the questions they were most interested in were not available. “I don’t think [the surgeons] could have answered the questions I could have asked. Like, ‘If we do surgery, I’ll have 100%; if we do closed reduction I’ll have X%?’ You know, those are the questions that I would want answers to”, explained a 71-year-old female. |
| Huetteman et al. | Patient factors | Facilitator | Knowledge and Communication | Internet as source for medical information | The information-seeking behavior described was limited to internet searches and consulting others who experienced a similar injury. These participants also explained that their professions (2 librarians and a researcher) drove them to pursue outside information. |
| Huetteman et al. | Patient factors | Barrier | Trust and Power | Trust towards healthcare personnel | A common theme among participants was taking comfort in the surgeon’s superior reputation (Table 7a). |
| Huetteman et al. | Patient factors | Barrier | Trust and Power | Trust towards healthcare personnel | When asked about the value they placed on the recommendation of the hand surgeon, 17 subjects explicitly stated that they valued it highly, expressing some variation on the sentiment, “The surgeon knows more than I do.” |
| Huetteman et al. | Decision-making interaction factors | Barrier | Knowledge and Communication | Knowledge/Competence asymmetry | When asked about the value they placed on the recommendation of the hand surgeon, 17 subjects explicitly stated that they valued it highly, expressing some variation on the sentiment, “The surgeon knows more than I do.” |
| Huetteman et al. | Patient factors | Barrier | Knowledge and Communication | Ease of non-involvment | When asked about the value they placed on the recommendation of the hand surgeon, 17 subjects explicitly stated that they valued it highly, expressing some variation on the sentiment, “The surgeon knows more than I do.” |
| Huetteman et al. | Patient factors | Barrier | Attitude and Behaviour | Depending on family or healthcare personnel | A final, but recurrent, point of influence for patients was the recommendations from a healthcare-affiliated friend or family member. When asked about the influence of others in the decision-making or information-seeking, multiple patients explained how they asked the advice of a friend or family member who worked in healthcare |
| Huetteman et al. | Patient factors | Facilitator | Attitude and Behaviour | Wanting to be involved through decisional participation | Despite some participants not showing strong preferences for a particular DRF treatment option, most subjects wanted a role in dictating specific features, like the type of anesthesia used or whether they underwent post-operative therapy with a therapist |
| Huetteman et al. | Patient factors | Facilitator | Trust and Power | Exercising power and dominance | Despite some participants not showing strong preferences for a particular DRF treatment option, most subjects wanted a role in dictating specific features, like the type of anesthesia used or whether they underwent post-operative therapy with a therapist |
| Huetteman et al. | Healthcare system /organisatonal factors | Facilitator | Health and Age | Need for individualized care - Treatment complexity and multimorbid patients | These patients indicated the need for individualized care. |
| Huetteman et al. | Patient factors | Facilitator | Knowledge and Communication | Wanting to express themselves, issue opinions and preferences and to be heard | Although patients did not necessarily share the decision for a treatment with their physician, many perceived that by providing their input on other factors, they still partook in shared decision-making |
| Huetteman et al. | Decision-making interaction factors | Barrier | Treatment Organization and Risk | Treatment related dismissal of decisional involvment | Nonetheless, we noticed a trend indicating that patients who have sustained conditions that they perceive to have been more severe than their DRF were less likely to want an active role |
| Huetteman et al. | Patient factors | Barrier | Treatment Organization and Risk | Treatment related dismissal of decisional involvment | Nonetheless, we noticed a trend indicating that patients who have sustained conditions that they perceive to have been more severe than their DRF were less likely to want an active role |
| Huetteman et al. | Decision-making interaction factors | Barrier | Knowledge and Communication | Knowledge/Competence asymmetry | Many participants described leaving the final decision in the hands of the specialist because of their own lack of knowledge and experience with such injuries. |
| Huetteman et al. | Patient factors | Barrier | Attitude and Behaviour | Depending on family or healthcare personnel | As seen in our study, individuals seek advice from others in the healthcare field, notwithstanding the outside source’s medical specialty. |
| Uldry et al. | Patient factors | Facilitator | Attitude and Behaviour | Wanting to be involved through decisional participation | In considering surgical complications or treatment in the intensive care unit, 64 % of patients wished to take an active role in any medical decisions |
| Uldry et al. | Patient factors | Facilitator | Attitude and Behaviour | Wanting to be informed or demanding more information | As for information, 73, 77, and 47 % of patients wish detailed information, information on a potential ICU hospitalization, and knowledge of cardiac resuscitation, respectively. |
| Uldry et al. | Patient factors | Facilitator | Attitude and Behaviour | Wanting to be informed or demanding more information | Thus, 72.7 % of the patients wished detailed information preoperatively (Q1), 31.6 % wished to be informed on all potential complications related to treatment (Q2), |
| Uldry et al. | Patient factors | Facilitator | Attitude and Behaviour | Active behaviour | Thus, 72.7 % of the patients wished detailed information preoperatively (Q1), 31.6 % wished to be informed on all potential complications related to treatment (Q2), |
| Uldry et al. | Patient factors | Facilitator | Attitude and Behaviour | Wanting to be involved through decisional participation | 76.7 % found it important prior to an operative intervention to have the opportunity to discuss the possible need for ICU admission (Q3), and 47.0 % found it essential to discuss what should be done in case of a cardiac arrest, which represents the worst case scenario during the postoperative course |
| Uldry et al. | Patient factors | Facilitator | Attitude and Behaviour | Active behaviour | 76.7 % found it important prior to an operative intervention to have the opportunity to discuss the possible need for ICU admission (Q3), and 47.0 % found it essential to discuss what should be done in case of a cardiac arrest, which represents the worst case scenario during the postoperative course |
| Uldry et al. | Patient factors | Facilitator | Attitude and Behaviour | Wanting to be involved through decisional participation | Thus, 63.6 % of all patients would like to actively participate in the medical decision making if a secondary operation becomes mandatory to treat complications (Q5), and 61.3 % in case a future ICU stay becomes necessary (Q6). |
| Uldry et al. | Patient factors | Facilitator | Attitude and Behaviour | Wanting to be involved through decisional participation | In the particular case of cancer treatment, 59.9 % of all patients judged it essential to discuss possible therapeutic limitations if severe, lifethreatening complications were to occur |
| Uldry et al. | Patient factors | Facilitator | Attitude and Behaviour | Wanting to be informed or demanding more information | Most patients were in favor of receiving detailed information about the planned operation and the postoperative course (e.g., ICU stay). |
| Uldry et al. | Patient factors | Facilitator | Attitude and Behaviour | Wanting to be involved through decisional participation | As long as patients are conscious and communication is preserved, they desire to be involved in all decision making, especially where important decisions must be made (e.g., reoperation, re-transfer to ICU, resuscitation). |
| Uldry et al. | Patient factors | Barrier | Attitude and Behaviour | Depending on family or healthcare personnel | Family members themselves become important if patients are unable to decide for themselves. |
| Uldry et al. | Patient factors | Barrier | Attitude and Behaviour | Passive behaviour | Elderly patients generally are less demanding of information, and it seems that they rely more on their physicians in the decision-making process. |
| Uldry et al. | Patient factors | Facilitator | Attitude and Behaviour | Wanting to be informed or demanding more information | Our study confirmed that the majority of patients are interested in getting specific preoperative information on the diagnosis, the planned surgical procedure, and the most frequent complications (73, 75, and 76 %, respectively). |
| Uldry et al. | Patient factors | Barrier | Attitude and Behaviour | Passive behaviour | Of note, elderly patients were significantly less interested in receiving extensive information. It is possible that these patients have cognitive impairments that limit their capacity to understand complex explanations; moreover, traditionally, preceding generations are used to trusting their doctors |
| Uldry et al. | Patient factors | Barrier | Trust and Power | Trust towards healthcare personnel | Of note, elderly patients were significantly less interested in receiving extensive information. It is possible that these patients have cognitive impairments that limit their capacity to understand complex explanations; moreover, traditionally, preceding generations are used to trusting their doctors |
| Uldry et al. | Patient factors | Facilitator | Attitude and Behaviour | Wanting to be informed or demanding more information | Patients were very eager to be informed about a possible ICU stay, since they probably interpreted it as a sign of severity of their disease and the planned surgery. |
| Uldry et al. | Patient factors | Barrier | Treatment Organization and Risk | Treatment related dismissal of decisional involvment | Likewise, if the intervention is judged to be minor, patients may assume that also the risks are limited and very detailed information is not needed. |
| Uldry et al. | Patient factors | Facilitator | Attitude and Behaviour | Wanting to be involved through decisional participation | Our study dedicated to surgical patients shows that patients strongly wish to be included in decision making during ongoing treatment |
| Uldry et al. | Patient factors | Facilitator | Attitude and Behaviour | Wanting to be involved through decisional participation | The vast majority of surgical patients clearly want to get adequate preoperative information about their disease and the planned treatment. They also consider it crucial to be involved in any kind of decision making. |
| Uldry et al. | Patient factors | Facilitator | Attitude and Behaviour | Wanting to be informed or demanding more information | The vast majority of surgical patients clearly want to get adequate preoperative information about their disease and the planned treatment. They also consider it crucial to be involved in any kind of decision making. |
| Uldry et al. | Patient factors | Facilitator | Attitude and Behaviour | Being enabled to ask questions and make decisions | Surgeons should avoid predicting their patients’ preferences and start providing patients with a climate of ‘‘open communication’’ that allows the patient to achieve the desired level of participation during decision making |
| Barrett et al. | Decision-making interaction factors | Barrier | Knowledge and Communication | Dominant communication semantics | Audiotaped encounters between primary care providers and patients with earlier stage CKD and CKD risk factors found that providers often use high levels of technical jargon and do not check patients’ comprehension |
| Barrett et al. | Healthcare personell factors | Barrier | Knowledge and Communication | Dominant communication semantics | Audiotaped encounters between primary care providers and patients with earlier stage CKD and CKD risk factors found that providers often use high levels of technical jargon and do not check patients’ comprehension |
| Barrett et al. | Decision-making interaction factors | Barrier | Trust and Power | Unknown healthcare provider | SDM discussions were more prevalent among participants who were under the care of their nephrologists for longer periods, completed more nephrology visits, and were at a high risk for kidney failure in the next 2 year. Even among those with a high risk for kidney failure, approximately a third had not discussed dialysis or transplantation. These findings highlight the importance of establishing longitudinal patient-provider relationships to promote SDM in kidney care, but they also show the need to enhance discussions, even in the context of longitudinal care. |
| Barrett et al. | Decision-making interaction factors | Facilitator | Trust and Power | Knowing healthcare provider / relationship | SDM discussions were more prevalent among participants who were under the care of their nephrologists for longer periods, completed more nephrology visits, and were at a high risk for kidney failure in the next 2 year. Even among those with a high risk for kidney failure, approximately a third had not discussed dialysis or transplantation. These findings highlight the importance of establishing longitudinal patient-provider relationships to promote SDM in kidney care, but they also show the need to enhance discussions, even in the context of longitudinal care. |
| Barrett et al. | Patient factors | Barrier | Attitude and Behaviour | Depending on family or healthcare personnel | However, our findings suggest that patients may not initiate SDM discussions simply because it is their preference. Rather, these patients may be waiting for their providers to engage them in such discussions, and kidney care teams should be aware that patients may not actively pursue their preferred decisionmaking style. |
| Barrett et al. | Patient factors | Barrier | Attitude and Behaviour | Passive behaviour | However, our findings suggest that patients may not initiate SDM discussions simply because it is their preference. Rather, these patients may be waiting for their providers to engage them in such discussions, and kidney care teams should be aware that patients may not actively pursue their preferred decisionmaking style. |
| Barrett et al. | Patient factors | Facilitator | Attitude and Behaviour | Active behaviour | In summary, most study participants preferred a shared approach to kidney treatment decision making |
| Barrett et al. | Decision-making interaction factors | Barrier | Treatment Organization and Risk | Lack of integration in social practices | Despite this preference, discussions were infrequent and were often incomplete when they occurred. |
| Barrett et al. | Decision-making interaction factors | Barrier | Treatment Organization and Risk | Lack of integration in social practices | Although most participants in our study preferred SDM, our results do not indicate that preference for SDM is associated with the actual occurrence of discussions. Prior studies also suggest that patients generally prefer SDM in other therapeutic areas. |
| De Roo et al. | Healthcare personell factors | Barrier | Treatment Organization and Risk | Time pressure | Other challenges in achieving shared decisionmaking included: time constraints, no preexisting relationship with patients, and the perceived ability of patients to understand. |
| De Roo et al. | Healthcare personell factors | Barrier | Trust and Power | Knowing healthcare provider / relationship | Other challenges in achieving shared decisionmaking included: time constraints, no preexisting relationship with patients, and the perceived ability of patients to understand. |
| De Roo et al. | Patient factors | Barrier | Knowledge and Communication | Lack of medical or treatment related knowledge | Other challenges in achieving shared decisionmaking included: time constraints, no preexisting relationship with patients, and the perceived ability of patients to understand. |
| De Roo et al. | Healthcare personell factors | Barrier | Treatment Organization and Risk | Treatment related dismissal of decisional involvment | Guiding patients at high-risk for harm may be particularly difficult because current evidence may not apply to this group, the consequences of nonoperative management may be unclear, and communicating uncertainty is challenging |
| De Roo et al. | Patient factors | Barrier | Knowledge and Communication | Knowledge/Competence asymmetry | Additionally, although the surgeons identified multiple obstacles to patient involvement, surgeons rarely discussed taking ownership to guide or educate patients. |
| De Roo et al. | Patient factors | Barrier | Attitude and Behaviour | Passive behaviour | Furthermore, patients were described as frequently deferring their role in decision-making, which may also indicate patients’ perceptions of and surgeons’ acceptance of the power differential in the surgeon-patient relationship regardless of the degree of shared decision-making |
| De Roo et al. | Healthcare system /organisatonal factors | Barrier | Treatment Organization and Risk | Lack of integration in social practices | Finally, because the shift to greater patient involvement has occurred after training for many of our participants, including patients in decision-making may represent a new challenge for practicing surgeons. During residency, communication training focuses primarily on delivering bad news |
| De Roo et al. | Healthcare system /organisatonal factors | Barrier | Treatment Organization and Risk | Lack of integration in social practices | Many trainees learn from senior surgeons and may not see shared decision-making modeled. How communication skills are taught, maintained, and evaluated in real practice is unclear and likely highly variable.28 Resources for improving patient involvement may include communication frameworks and education from other physicians with expertise in communication. |
| De Roo et al. | Healthcare personell factors | Barrier | Attitude and Behaviour | No decisional involvment of patients | Additionally, some surgeons may believe the current state is adequate and does not require further patient involvement. |
| De Roo et al. | Healthcare system /organisatonal factors | Facilitator | Health and Age | Need for individualized care - Treatment complexity and multimorbid patients | When there was an apparent mismatch between patient goals and surgeon expectations, participating surgeons described shared decision-making and reaching an agreement together with the patient. |
| De Roo et al. | Healthcare system /organisatonal factors | Barrier | Treatment Organization and Risk | Lack of integration in social practices | One potential reason for the absence was the relatively recent change to emphasize patient involvement in decision-making. |
| Verberne et al. | Patient factors | Facilitator | Treatment Organization and Risk | Satisfying involvment | The majority reported to be satisfied with their decision-making process and treatment choice. |
| Verberne et al. | Patient factors | Facilitator | Treatment Organization and Risk | Treatment satisfaction | The majority reported to be satisfied with their decision-making process and treatment choice. |
| Verberne et al. | Patient factors | Barrier | Treatment Organization and Risk | Timely treatment necessity | Those answering negatively would have preferred more time to consider their situation more extensively, to prepare themselves better: “[Counselling] should have started much earlier, I was shocked when I heard about it [dialysis]. The doctor should have acted more in advance” (dialysis patient), |
| Verberne et al. | Patient factors | Barrier | Treatment Organization and Risk | Treatment related dismissal of decisional involvment | although some acknowledged that their clinical condition restricted time for decision making. |
| Verberne et al. | Healthcare personell factors | Barrier | Treatment Organization and Risk | Treatment related dismissal of decisional involvment | although some acknowledged that their clinical condition restricted time for decision making. |
| Verberne et al. | Patient factors | Barrier | Trust and Power | Feeling powerless / Having no control | One third of the patients who had chosen dialysis reported they had felt forced to make a decisio |
| Verberne et al. | Healthcare personell factors | Barrier | Trust and Power | Exercising power and dominance | One third of the patients who had chosen dialysis reported they had felt forced to make a decisio |
| Verberne et al. | Decision-making interaction factors | Barrier | Trust and Power | Asymmetric power relationship | One third of the patients who had chosen dialysis reported they had felt forced to make a decisio |
| Verberne et al. | Patient factors | Barrier | Trust and Power | Asymmetric power relationship | One third of the patients who had chosen dialysis reported they had felt forced to make a decisio |
| Verberne et al. | Healthcare personell factors | Barrier | Trust and Power | Asymmetric power relationship | One third of the patients who had chosen dialysis reported they had felt forced to make a decisio |
| Verberne et al. | Healthcare personell factors | Barrier | Trust and Power | Treatment related dismissal of decisional involvment | Most patients mentioned to have felt forced due to the circumstances, such as deteriorating health or kidney function, or by their nephrologist |
| Verberne et al. | Healthcare personell factors | Barrier | Trust and Power | Exercising power and dominance | Most patients mentioned to have felt forced due to the circumstances, such as deteriorating health or kidney function, or by their nephrologist |
| Verberne et al. | Patient factors | Barrier | Attitude and Behaviour | Not having a choice | Some mentioned their relatives, or a perceived lack of choice |
| Verberne et al. | Patient factors | Barrier | Attitude and Behaviour | Not having a choice | One CC patient answered that treatment options other than CC were barely discussed |
| Verberne et al. | Healthcare personell factors | Barrier | Knowledge and Communication | No treatment or information involvment of patients | One CC patient answered that treatment options other than CC were barely discussed |
| Verberne et al. | Patient factors | Facilitator | Treatment Organization and Risk | Satisfying involvment | The majority reported they had experienced sufficient guidance from the healthcare team during decision making |
| Verberne et al. | Healthcare personell factors | Barrier | Treatment Organization and Risk | Time pressure | Those who experienced insufficient guidance mentioned they needed more time and information. One patient an swered: “In retrospect, I did not completely understand what dialysis is. An educational video using simple and comprehensible language would have been nice. Particularly because of [my] reduced capacity to process information due to my high age” (CC patient). |
| Verberne et al. | Healthcare personell factors | Barrier | Knowledge and Communication | No treatment or information involvment of patients | Those who experienced insufficient guidance mentioned they needed more time and information. One patient an swered: “In retrospect, I did not completely understand what dialysis is. An educational video using simple and comprehensible language would have been nice. Particularly because of [my] reduced capacity to process information due to my high age” (CC patient). |
| Verberne et al. | Patient factors | Barrier | Knowledge and Communication | Lack of medical or treatment related knowledge | Those who experienced insufficient guidance mentioned they needed more time and information. One patient an swered: “In retrospect, I did not completely understand what dialysis is. An educational video using simple and comprehensible language would have been nice. Particularly because of [my] reduced capacity to process information due to my high age” (CC patient). |
| Verberne et al. | Healthcare personell factors | Barrier | Knowledge and Communication | Linguistic issues | Those who experienced insufficient guidance mentioned they needed more time and information. One patient an swered: “In retrospect, I did not completely understand what dialysis is. An educational video using simple and comprehensible language would have been nice. Particularly because of [my] reduced capacity to process information due to my high age” (CC patient). |
| Verberne et al. | Patient factors | Barrier | Health and Age | Being old | Those who experienced insufficient guidance mentioned they needed more time and information. One patient an swered: “In retrospect, I did not completely understand what dialysis is. An educational video using simple and comprehensible language would have been nice. Particularly because of [my] reduced capacity to process information due to my high age” (CC patient). |
| Verberne et al. | Patient factors | Barrier | Health and Age | Being overstrained | Those who experienced insufficient guidance mentioned they needed more time and information. One patient an swered: “In retrospect, I did not completely understand what dialysis is. An educational video using simple and comprehensible language would have been nice. Particularly because of [my] reduced capacity to process information due to my high age” (CC patient). |
| Verberne et al. | Healthcare personell factors | Barrier | Knowledge and Communication | No treatment or information involvment of patients | Suggested improvements were: more information on all treatment options, tailoring of information to an individual’s situation, and more time, deliberation, and involvement in decision-making. |
| Verberne et al. | Patient factors | Barrier | Attitude and Behaviour | Not having a choice | Suggested improvements were: more information on all treatment options, tailoring of information to an individual’s situation, and more time, deliberation, and involvement in decision-making. |
| Verberne et al. | Healthcare personell factors | Barrier | Knowledge and Communication | Linguistic issues | Suggested improvements were: more information on all treatment options, tailoring of information to an individual’s situation, and more time, deliberation, and involvement in decision-making. |
| Verberne et al. | Healthcare personell factors | Barrier | Knowledge and Communication | No decisional involvment of patients | Suggested improvements were: more information on all treatment options, tailoring of information to an individual’s situation, and more time, deliberation, and involvement in decision-making. |
| Verberne et al. | Healthcare personell factors | Barrier | Treatment Organization and Risk | Time pressure | Suggested improvements were: more information on all treatment options, tailoring of information to an individual’s situation, and more time, deliberation, and involvement in decision-making. |
| Verberne et al. | Patient factors | Barrier | Attitude and Behaviour | Not having a choice | patients who had chosen dialysis most frequently mentioned a perceived lack of choice |
| Verberne et al. | Healthcare personell factors | Barrier | Knowledge and Communication | No treatment or information involvment of patients | However, we observed a discrepancy between the high satisfaction and underlying negative experiences that older patients reported as well, especially patients who had chosen dialysis. Such negative experiences were related to the timing, informing, and level of decision making being shared. |
| Verberne et al. | Healthcare personell factors | Barrier | Treatment Organization and Risk | Time pressure | However, we observed a discrepancy between the high satisfaction and underlying negative experiences that older patients reported as well, especially patients who had chosen dialysis. Such negative experiences were related to the timing, informing, and level of decision making being shared. |
| Verberne et al. | Healthcare personell factors | Barrier | Knowledge and Communication | No decisional involvment of patients | However, we observed a discrepancy between the high satisfaction and underlying negative experiences that older patients reported as well, especially patients who had chosen dialysis. Such negative experiences were related to the timing, informing, and level of decision making being shared. |
| Verberne et al. | Patient factors | Barrier | Attitude and Behaviour | Not having a choice | we found indications that decision-making should be initiated earlier because older patients felt unprepared or even forced to decide. |
| Verberne et al. | Patient factors | Barrier | Trust and Power | Feeling powerless / Having no control | we found indications that decision-making should be initiated earlier because older patients felt unprepared or even forced to decide. |
| Verberne et al. | Healthcare personell factors | Barrier | Trust and Power | Exercising power and dominance | we found indications that decision-making should be initiated earlier because older patients felt unprepared or even forced to decide. |
| Verberne et al. | Decision-making interaction factors | Barrier | Trust and Power | Asymmetric power relationship | we found indications that decision-making should be initiated earlier because older patients felt unprepared or even forced to decide. |
