# Supplementary material for: Patients’ and healthcare professionals’ perceived facilitators and barriers for shared decision-making for frail and elderly patients in perioperative care: a scoping review
Source: BMC Health Serv Res. 2023 Feb 24;23:197. doi: 10.1186/s12913-023-09120-4 (PMC9960423; doi:10.1186/s12913-023-09120-4)
Supplement: Supplementary file 5 — Additional file 5: Appendix 5. Collated Categories, Subcategories and Stakeholders. [file 12913_2023_9120_MOESM5_ESM.docx]

**Appendix 5: Collated Categories, Subcategories and Stakeholders**

| **Category** | **Subcategory** | **Stakeholders** | | | | | | |
| --- | --- | --- | --- | --- | --- | --- | --- | --- |
|  |  |  |  |  |  |  |  |  |
| **Attitude and behavior** | Active behavior |  |  |  |  |  |  |  |
|  |  |  |  |  |  |  |  |  |
|  | Wanting to be involved through decisional participation |  |  |  |  |  |  |  |
|  |  |  |  |  |  |  |  |  |
|  | Wanting to be informed or demanding more information |  |  |  |  |  |  |  |
|  |  |  |  |  |  |  |  |  |
|  | Depending on family or healthcare personnel |  |  |  |  |  |  |  |
|  |  |  |  |  |  |  |  |  |
|  | Passive behavior |  |  |  |  |  |  |  |
|  |  |  |  |  |  |  |  |  |
|  | No decisional involvement of patients |  |  |  |  |  |  |  |
|  |  |  |  |  |  |  |  |  |
|  | Not wanting to participate in decision making |  |  |  |  |  |  |  |
|  |  |  |  |  |  |  |  |  |
|  | No treatment or information involvement of patients |  |  |  |  |  |  |  |
|  |  |  |  |  |  |  |  |  |
|  | Submissive behavior |  |  |  |  |  |  |  |
|  |  |  |  |  |  |  |  |  |
|  | Confidence in participating in decisional involvement |  |  |  |  |  |  |  |
|  |  |  |  |  |  |  |  |  |
|  | Not having a choice |  |  |  |  |  |  |  |

| **Category** | **Subcategory** | **Stakeholders** | | | | | | | | | | | | |  |  |
| --- | --- | --- | --- | --- | --- | --- | --- | --- | --- | --- | --- | --- | --- | --- | --- | --- |
|  |  |  | |  | |  | | |  |  | | |  |  |  |  |
| **Trust and Power** | Exercising power and dominance |  |  | |  | |  |  | | |  |  | | | |  |
|  |  |  |  | |  | |  |  | | |  |  | | | |  |
|  | Trust towards healthcare personnel |  |  | |  | |  |  | | |  |  | | | |  |
|  |  |  |  | |  | |  |  | | |  |  | | | |  |
|  | Unknown healthcare provider |  |  | |  | |  |  | | |  |  | | | |  |
|  |  |  |  | |  | |  |  | | |  |  | | | |  |
|  | Asymmetric power relationship and dominance |  |  | |  | |  |  | | |  |  | | | |  |
|  |  |  |  | |  | |  |  | | |  |  | | | |  |
|  | Feeling incapacitated |  |  | |  | |  |  | | |  |  | | | |  |
|  |  |  |  | |  | |  |  | | |  |  | | | |  |
|  | Feeling powerless / Having no control |  |  | |  | |  |  | | |  |  | | | |  |
|  |  |  |  | |  | |  |  | | |  |  | | | |  |
|  | Submissive communication semantics |  |  | |  | |  |  | | |  |  | | | |  |
|  |  |  |  | |  | |  |  | | |  |  | | | |  |
|  | Fear of incompliance |  |  | |  | |  |  | | |  |  | | | |  |
|  |  |  |  | |  | |  |  | | |  |  | | | |  |
|  | Feeling controlled |  |  | |  | |  |  | | |  |  | | | |  |
|  |  |  |  | |  | |  |  | | |  |  | | | |  |
|  | Institution of power and/or trust |  |  | |  | |  |  | | |  |  | | | | |

|  |  |  | | | | | | |
| --- | --- | --- | --- | --- | --- | --- | --- | --- |
|  |  |  | | | | | | |
| **Category** | **Subcategory** | **Stakeholders** | | | | | | |
|  |  |  |  |  |  |  |  |  |
| **Knowledge and Communication** | Wanting to be involved through decisional participation |  |  |  |  |  |  |  |
|  |  |  |  |  |  |  |  |  |
|  | Wanting to be informed or demanding more information |  |  |  |  |  |  |  |
|  |  |  |  |  |  |  |  |  |
|  | Wanting to express themselves, issue opinions and preferences and to be heard |  |  |  |  |  |  |  |
|  |  |  |  |  |  |  |  |  |
|  | Depending on family or healthcare personnel |  |  |  |  |  |  |  |
|  |  |  |  |  |  |  |  |  |
|  | Knowledge/Competence asymmetry |  |  |  |  |  |  |  |
|  |  |  |  |  |  |  |  |  |
|  | No treatment or information involvement of patients |  |  |  |  |  |  |  |
|  |  |  |  |  |  |  |  |  |
|  | Lack of medical or treatment related knowledge |  |  |  |  |  |  |  |
|  |  |  |  |  |  |  |  |  |
|  | Internet as source for medical information |  |  |  |  |  |  |  |
|  |  |  |  |  |  |  |  |  |
|  | Medical knowledge is not required |  |  |  |  |  |  |  |
|  |  |  |  |  |  |  |  |  |
|  | Linguistic issues |  |  |  |  |  |  |  |
|  |  |  |  |  |  |  |  |  |
|  | Dominant communication semantics |  |  |  |  |  |  |  |
|  |  |  |  |  |  |  |  |  |
|  | Submissive communication semantics |  |  |  |  |  |  |  |
|  |  |  |  |  |  |  |  |  |

| **Knowledge and Communication** | Adequate medical knowledge |  |  |  |  |  |  |  |
| --- | --- | --- | --- | --- | --- | --- | --- | --- |
|  |  |  |  |  |  |  |  |  |
|  | Being offered a choice |  |  |  |  |  |  |  |
|  |  |  |  |  |  |  |  |  |
|  | Supporting family involvement |  |  |  |  |  |  |  |
|  |  |  |  |  |  |  |  |  |
|  | Ease of non-involvement |  |  |  |  |  |  |  |
|  |  |  |  |  |  |  |  |  |
|  | Diverging perceptions of health condition, treatment or surgical outcome |  |  |  |  |  |  |  |
|  |  |  |  |  |  |  |  |  |
|  | Prior misinformation through family, friends, internet or other sources |  |  |  |  |  |  |  |

| **Category** | **Subcategory** | **Stakeholders** | | | | | | |
| --- | --- | --- | --- | --- | --- | --- | --- | --- |
|  |  |  |  |  |  |  |  |  |
| **Treatment Organization and Risk** | Treatment related dismissal of decisional involvement |  |  |  |  |  |  |  |
|  |  |  |  |  |  |  |  |  |
|  | Time pressure |  |  |  |  |  |  |  |
|  |  |  |  |  |  |  |  |  |
|  | High workload |  |  |  |  |  |  |  |
|  |  |  |  |  |  |  |  |  |
|  | Alternative choices / Ambiguity |  |  |  |  |  |  |  |
|  |  |  |  |  |  |  |  |  |
|  | Healthcare staff rotation |  |  |  |  |  |  |  |
|  |  |  |  |  |  |  |  |  |
|  | Lack of integration in social practices |  |  |  |  |  |  |  |
|  |  |  |  |  |  |  |  |  |
|  | SDM mediator |  |  |  |  |  |  |  |
|  |  |  |  |  |  |  |  |  |
|  | Adequate workload |  |  |  |  |  |  |  |
|  |  |  |  |  |  |  |  |  |
|  | Formal SDM approach |  |  |  |  |  |  |  |
|  |  |  |  |  |  |  |  |  |
|  | Timely treatment necessity |  |  |  |  |  |  |  |
|  |  |  |  |  |  |  |  |  |
|  | Facing diverging treatment strategies |  |  |  |  |  |  |  |
|  |  |  |  |  |  |  |  |  |
|  | Acute setting |  |  |  |  |  |  |  |
|  |  |  |  |  |  |  |  |  |
|  | Patient turnover |  |  |  |  |  |  |  |
|  |  |  |  |  |  |  |  |  |
|  | Scheduling issue |  |  |  |  |  |  |  |

| **Category** | **Subcategory** | **Stakeholders** | | | | | | | |
| --- | --- | --- | --- | --- | --- | --- | --- | --- | --- |
|  |  |  |  |  |  |  |  |  |  |
| **Health and Age** | Being old |  |  |  |  |  |  |  |  |
|  |  |  |  |  |  |  |  |  |  |
|  | Being ill |  |  |  |  |  |  |  |  |
|  |  |  |  |  |  |  |  |  |  |
|  | Being in pain |  |  |  |  |  |  |  |  |
|  |  |  |  |  |  |  |  |  |  |
|  | Being overstrained |  |  |  |  |  |  |  |  |
|  |  |  |  |  |  |  |  |  |  |
|  | Forgetting discussions or given information |  |  |  |  |  |  |  |  |
|  |  |  |  |  |  |  |  |  |  |
|  | Need for individualized care - Treatment complexity and multimorbid patients |  |  |  |  |  |  |  |  |
|  |  |  |  |  |  |  |  |  |  |
|  | Being confused |  |  |  |  |  |  |  |  |
|  |  |  |  |  |  |  |  |  |  |
|  | Being tired |  |  |  |  |  |  |  |  |
|  |  |  |  |  |  |  |  |  |  |
|  | Timely treatment necessity |  |  |  |  |  |  |  |  |
