# Supplementary material for: Patients’ and healthcare professionals’ perceived facilitators and barriers for shared decision-making for frail and elderly patients in perioperative care: a scoping review
Source: BMC Health Serv Res. 2023 Feb 24;23:197. doi: 10.1186/s12913-023-09120-4 (PMC9960423; doi:10.1186/s12913-023-09120-4)
Supplement: Supplementary file 6 — Additional file 6: Appendix 6. Taxonomy of barriers and facilitators. [file 12913_2023_9120_MOESM6_ESM.docx]

| **Appendix 6 Taxonomy of barriers and facilitators** | | | | |
| --- | --- | --- | --- | --- |
| **Category** | **Subcategory** | **Definition** | **No Articles** | **% total** |
| Attitude and Behavior | Active behavior | Patients assume an active part in the discussions and decision-making process | 9 | 69% |
|  | Wanting to be involved through decisional participation | Patients want to participate in decision-making procedures. | 9 | 69% |
|  | Wanting to be informed or demanding more information | Patients want to be informed and demand information actively. | 7 | 54% |
|  | Depending on family or healthcare personnel | Patients depend on family members or clinical staff for decision-making. | 6 | 46% |
|  | Passive behavior | Patients need assistance from family members or clinical staff for treatment-related discussions and decisions | 5 | 38% |
|  | No decisional involvement of patients | Patients are not involved in decision-making processes by healthcare professionals | 5 | 38% |
|  | Not wanting to participate in decision making | Patients do not want to assume an active part in the discussions and decision-making process | 4 | 31% |
|  | No treatment or information involvement of patients | Patients are not informed or involved during discussions and decisions about subsequent treatments by healthcare professionals. | 4 | 31% |
|  | Not having a choice | Patients believe that they have no choice. | 4 | 31% |
|  | Submissive behavior | Patients exhibit submissive behavior towards healthcare professionals in the discussions and decision-making process | 3 | 23% |
|  | Confidence in participating in decisional involvement | Patients are confident about their participation in treatment related discussions and decision-making processes | 3 | 23% |
|  | Being enabled to ask questions and make decisions | Patients feel they are heard, consulted, and involved in the discussions and decision-making process | 2 | 15% |

| **Category** | **Subcategory** | **Definition** | **No Articles** | **% total** |
| --- | --- | --- | --- | --- |
| Knowledge and Communication | Wanting to be involved through decisional participation | Patients want to participate in decision-making procedures. | 9 | 69% |
|  | Wanting to be informed or demanding more information | Patients want to be informed and demand information actively. | 7 | 54% |
|  | Wanting to express themselves, issue opinions and preferences and to be heard | Patients want to participate in decision-making processes, express their opinions and be heard. | 6 | 46% |
|  | Depending on family or healthcare personnel | Patients depend on family members or clinical staff for decision-making. | 6 | 46% |
|  | No treatment or information involvement of patients | Patients are not informed or involved during discussions and decisions about subsequent treatments by healthcare professionals. | 5 | 38% |
|  | Knowledge/Competence asymmetry | Asymmetries of knowledge and competence impede joint participation in discussions and decision-making. | 4 | 31% |
|  | Lack of medical or treatment related knowledge | Patients lack sufficient medical knowledge and know-how of subsequent treatment. | 4 | 31% |
|  | Linguistic issues | Language barriers, on the part of patients, are an obstacle to participation in discussions and decision-making process | 3 | 23% |
|  | Internet as source for medical information | Patients obtain information about their condition and treatment options online, on their own. | 2 | 15% |
|  | Medical knowledge is not required | Patients or healthcare professionals consider that medical knowledge is not the basis for participation. | 2 | 15% |
|  | Dominant communication semantics | Healthcare professionals use dominant and incomprehensible language when speaking to patients. | 2 | 15% |
|  | Submissive communication semantics | Patients communicate in a submissive manner and wording. | 2 | 15% |
|  | Adequate medical knowledge | Patients have or attribute to themselves a level of medical knowledge appropriate for participation in discussions and decision-making process | 1 | 8% |
|  | Being offered a choice | Patients are presented with decision options. | 1 | 8% |
|  | Supporting family involvement | Patients have a supportive environment. | 1 | 8% |
|  | Ease of non-involvement | Patients find it easier not to be involved in discussions and decision-making processes. | 1 | 8% |
|  | Diverging perceptions of health condition, treatment or surgical outcome | The understanding of the health condition, treatment options, and potential outcomes varies among participating stakeholders. | 1 | 8% |
|  | Prior misinformation through family, friends, internet or other sources | Patients were misinformed regarding their condition, symptoms, or treatment options before being admitted to the clinic. | 1 | 8% |

| **Category** | **Subcategory** | **Definition** | **No Articles** | **% total** |
| --- | --- | --- | --- | --- |
| Trust and Power | Exercising power and dominance | Healthcare professionals or patients exhibit dominant behavior towards their counterpart. | 6 | 46% |
|  | Trust towards healthcare personnel | Patients trust healthcare professionals | 4 | 31% |
|  | No treatment or information involvement of patients | Patients are not informed or involved during discussions and decisions about subsequent treatments by healthcare professionals. | 4 | 31% |
|  | Asymmetric power relationship and dominance | Asymmetries of power and dominance impede joint participation in discussions and decision-making. | 4 | 27% |
|  | Unknown healthcare provider | Patients do not know the healthcare professional or physician in charge of their care | 3 | 23% |
|  | Feeling powerless / Having no control | Patients perceive that they have no decision-making power or control in the discussions and decision-making process and treatment | 3 | 23% |
|  | Submissive behavior | Patients exhibit submissive behavior towards healthcare professionals in the discussions and decision-making process | 3 | 23% |
|  | Feeling incapacitated | Patients feel incapacitated in the clinical setting or specifically in the discussions and decision-making process | 2 | 15% |
|  | Submissive communication semantics | Patients communicate in a submissive manner and wording. | 2 | 15% |
|  | Fear of incompliance | Patients are afraid of negative consequences on their treatment, by healthcare professionals, if they do not follow their opinion | 1 | 8% |
|  | Feeling controlled | Patients feel controlled in the clinical setting or specifically in the discussions and decision-making process | 1 | 8% |
|  | Institution of power and/or trust | Patients trust the clinic and perceive it as a powerful institution | 1 | 8% |

| **Category** | | **Subcategory** | | **Definition** | | **No Articles** | | **% total** | |
| --- | --- | --- | --- | --- | --- | --- | --- | --- | --- |
| Treatment Organization and Risk | | Treatment related dismissal of decisional involvement | | Patients are not involved in discussions and decision-making processes; due to the treatment they are likely to receive | | 4 | | 31% | |
|  |  | Time pressure | | The healthcare professionals are under time pressure | | 4 | | 31% | |
|  |  | High workload | | The healthcare professionals have a high workload | | 3 | | 23% | |
|  |  | Satisfying involvement | | Patients are satisfied with participation in the discussions and decision-making process | | 3 | | 23% | |
|  |  | Treatment satisfaction | | Patients are satisfied with the treatment process | | 3 | | 23% | |
|  |  | Alternative choices / Ambiguity | | Patients can cope well with different treatment options, even if they are ambiguous | | 2 | | 15% | |
|  |  | Healthcare staff rotation | | Rotating clinical staff is a constraint to continuity of care and involving patients in discussions and decision-making processes | | 2 | | 15% | |
|  |  | Lack of integration in social practices | | Participatory decision-making processes and interactions between clinical staff and patients are not integrated into clinical processes | | 2 | | 15% | |
|  |  | Timely treatment necessity | | Patients are of the impression that the treatment must be done promptly | | 2 | | 15% | |
|  |  | Satisfying information sharing | | Patients are satisfied with the information shared | | 2 | | 15% | |
|  |  | SDM mediator | | SDM mediators, who assume no decision-making duties, may act as mediators between patients and healthcare professionals | | 1 | | 8% | |
|  |  | Adequate workload | | The healthcare professionals have an adequat workload | | 1 | | 8% | |
|  |  | Formal SDM approach | | A formal SDM approach supports implementation and success of participatory discussions and decision-making processes | | 1 | | 8% | |
|  |  | Facing different treatment strategies | | Patients continuously face different treatment options | | 1 | | 8% | |
|  |  | Acute setting | | Acute or emergency situations and decisions are inappropriate for participatory decision-making processes | | 1 | | 8% | |
|  |  | Patient turnover | | Patients attend the clinic only for a short time, impeding participatory discussions and decision-making processes | | 1 | | 8% | |
|  |  | Scheduling issue | | Scheduling issues for the clinic and individual health professions impede participatory discussions and decision-making processes | | 1 | | 8% | |
|  | | Diminution of decisional conflict | | Decision conflicts are minimized through discussions and decision-making process | | 1 | | 8% | |
| **Category** | **Subcategory** | | **Definition** | | **No Articles** | | **% total** | |  |
| Health and Age | Being old | | Patients are too old to be involved in discussions and decision-making processes. | | 4 | | 31% | |  |
|  | Being overstrained | | Patients are overburdened by being involved in discussions and decision-making processes. | | 3 | | 23% | |  |
|  | Being ill | | Patients are too restricted by their health condition to be involved in discussions and decision-making processes. | | 2 | | 15% | |  |
|  | Being in pain | | Patients feel too much pain to be involved in discussions and decision-making processes. | | 2 | | 15% | |  |
|  | Forgetting discussions or given information | | Patients forget the content and outcome of the discussions and decision-making processes. | | 2 | | 15% | |  |
|  | Need for individualized care - Treatment complexity and multimorbid patients | | Comorbid patients are at higher risk for severe postoperative outcomes and need individualized care | | 2 | | 15% | |  |
|  | Being confused | | Patients are confused by being involved in discussions and decision-making processes. | | 1 | | 8% | |  |
|  | Being tired | | Patients are too tired to be involved in discussions and decision-making processes. | | 1 | | 8% | |  |
|  | Timely treatment necessity | | Patients are of the impression that the treatment must be done promptly | | 1 | | 8% | |  |
|  | Discomfort due to too much involvement | | Patients experience discomfort due to over-commitment in discussions and decision-making process | | 1 | | 8% | |  |
